# Associations between long-term care-service use and service- or care-need level progression: a nationwide cohort study using the Japanese Long-Term Care Insurance Claims database

**DOI:** 10.1186/s12913-023-09615-0

**Published:** 2023-06-05

**Authors:** Kohei Hasegawa, Teruomi Tsukahara, Tetsuo Nomiyama

**Affiliations:** 1 Department of Preventive Medicine and Public Health, Shinshu University School of Medicine, 3-1-1 Asahi, Matsumoto, Nagano, 390-8621 Japan; 2 Department of Occupational Medicine, Shinshu University School of Medicine, 3-1-1 Asahi, Matsumoto, Nagano, 390-8621 Japan

**Keywords:** Long-term care insurance, Health service

## Abstract

**Background:**

The effectiveness of the long-term care service in Japan has been unclear, and most of the relevant studies of this service have been limited to a single region and relatively small samples, necessitating large-scale studies. We examined the associations between long-term care service use and the service/care-need level progression at the national scale in Japan.

**Methods:**

We conducted a nationwide retrospective cohort study using data from the Japanese Long-Term Care Insurance Claims database. Individuals aged ≥ 65 years and newly certified as being at the support-need level 1 or 2 or the care-need level 1 between April 2012 and March 2013 were included. We first conducted 1:1 propensity score matching and then examined the associations between service use and the progression in support-need or care-need levels by using Kaplan–Meier survival curves and log-rank tests.

**Results:**

The final sample consisted of 332,766 individuals. We observed that service use was associated with a faster decline in the support/care-need level, although the differences in the subjects' survival rate diminished; the log-rank test showed significance (*p* < 0.001). When stratified for urban–rural classifications or regions of Japan, the results were similar to the primary analysis in all of the stratified groups, and no clear regional variations were observed.

**Conclusion:**

We did not observe a clear beneficial effect of receiving long-term care in Japan. Our results suggest that Japan's current long-term care service may not be effective for the recipients of these services. Considering that the system is becoming a financial burden, a re-examination of the service to provide more cost-effective care may be advisable.

**Supplementary Information:**

The online version contains supplementary material available at 10.1186/s12913-023-09615-0.

## Background

Population aging is a worldwide trend, and the increasing number of elderly who need support or care has become a vital public health issue. Japan's long-term care insurance system for the elderly was introduced in 2000 to tackle this issue. In 2021, 28.9% of Japan's population was aged ≥ 65 years [1]. Under the long-term care insurance system, elders certified as needing support or care can receive long-term care with a 10% co-payment. However, as the number of recipients has rapidly increased, the system's financial burden has became untenably high [2], and the cost-effectiveness of the service is gaining more attention, especially among policymakers.

However, the effectiveness of the care provided by Japan's long-term care insurance system for the elderly has not been established. A number of studies were conducted, but their results are not consistent [3–7]. Each of these studies was conducted in a single region and the sample sizes were relatively small, which may partly explain the inconsistent results. Large-scale or nationwide studies have been desired, but such research has been limited. A nationwide study of Japan's long-term care insurance system was conducted recently, but it focused only on improvement effects that were documented in an approx. 6-month period, and the long-term effects have remained uncertain [8]. Japan's Ministry of Health, Labour and Welfare (MHLW) started collecting anonymized data on the long-term care insurance system in 2010 from nearly all insurers and then developed a nationwide database called the Japanese Long-Term Care Insurance Claims database [9, 10].

We conducted the present study to examine the associations between the use of Japan's long-term care service and the progression of service/care-need levels. Using the data from the above-mentioned nationwide database, we investigated the association at the national scale with a much larger sample size compared to previous studies.

## Methods

### Data source

We used de-identified certification data of long-term care and long-term care insurance claim data from the Japanese Long-Term Care Insurance Claims database, which is managed by the MHLW. Detailed descriptions of the Japanese long-term care insurance system and the database are provided elsewhere [3, 9–12]. In short, residents of Japan aged ≥ 65 (or 40–64 with specified diseases) are insured in Japan's long-term care insurance system. The system's insurers are the local (i.e., municipal) governments, but all of the procedures in the system are standardized. When insured individuals need long-term care, they make a request for the care at their local governmental offices. The insurer then conducts a certification survey to examine the individual's health status and care needs. Individuals who are certified as needing help in the certification survey can use the system's care services with a 10% co-payment based on their certified seven health status levels: two support-need levels (1 and 2) and five care-need levels (1 to 5), in which a higher level-number indicates a more dependent status. The services provided by the system can be broadly divided into two groups: home-based services and facility services [3, 13]. Home-based services aim to help individuals continue their daily life in their homes, and the services include home visiting services, daycare, short-stay services, and more. Facility services offer residential care to individuals who cannot continue to live at home. In principle, a certified individual's health status is re-examined within 6 months after the first certification and every 12 months after that. The MHLW collects the results of the certification survey and long-term care insurance claim data from almost all of the insurers throughout Japan and stores the data in the Japanese Long-Term Care Insurance Claims database.

The data that we obtained from the database included individual-level information from the survey results and claims data including age, sex, support-need or care-need level, physical and mental status, the medical care received in the past, the individual's insurer, and his or her long-term care service use. After an initial review of the data, we included individuals aged ≥ 65 years who were newly certified as being at support-need level 1 or 2 or care-need level 1 during the period from April 2012 through March 2013. We excluded individuals with a follow-up period ≤ 6 months and individuals with progression in their support/care-need level during the above-mentioned timeframe. We also excluded individuals living in long-term care facilities at the time of their certification (e.g., a nursing home, medical care facility, and similar establishments) in order to prevent the inclusion of previous users. We also excluded individuals who had used facility services within the first 6 months after their certification. To eliminate the cases of "social hospitalization," where individuals resort to hospital admission as a substitute for long-term care service for financial reasons [14], we further excluded individuals who were at medical intuitions at their certification. As a result, our final sample consisted of only individuals who were residing at home at the time of their certification survey.

Briefly, individuals at support-need level 1 can perform the majority of activities of daily living (ADLs) (e.g., walking, rising) independently but may need some assistance for instrumental ADLs such as taking oral medication, cooking, and shopping. Those with support-need level 2 have a slightly lower capacity to perform instrumental ADLs compared to individuals at support-need level 1. Individuals at care-need level 1 have difficulties performing ADLs alone and demonstrate even further reduced capacity to undertake instrumental ADLs, requiring occasional long-term care [15]. The support/care-need level is reported to be correlated with the Barthel index score, an established measurement of everyday living activities, ranging from 0 (complete dependence) to 100 (complete independence). The support-need level 1, support-need level 2, and care-need level 1 roughly correspond to Barthel index scores at 95, 90, and 85, respectively [16].

### Variables

The outcome of this study was progression in a support- or care-need level, as has been examined in similar studies [3–6, 10, 17, 18]. In the present study, we defined progression as an increase of ≥ 1 in a support- or care-need level. The exposure of interest was the utilization of preventive care services. We defined the exposed group (users) as individuals who used long-term care services within the initial 6 months after their certification [4], including the month of the individual's certification, as ascertained through claims data for long-term care services in the database. Of note, as we excluded individuals who used any facility services within the first 6 months, those in the exposure group used only home-based services. We defined the control group (non-users) as individuals who did not use any preventive care service during the same period.

We extracted the following individual characteristics from each individual's first certification record for long-term care service: sex (female and male), age as 5-year age groups (< 75, 75–79, 80–84, and ≥ 85), support- and care-need level(s), paralysis (yes or no), contractures (yes or no), a cognitive disorder (yes or no), a mental or behavioral disorder (yes or no), medical care (yes or no), and the insurer. We defined the presence of paralysis, contractures, cognitive disorders, mental or behavioral disorders, and receiving medical care as described [8]. Specifically, an individual was deemed to have paralysis or contractures if any manifestation of such a condition was present in any region of their body. We defined cognitive disorder as the inability to perform any of the seven cognitive functions: conveying intentions to others, understanding the daily routine, recalling one's date of birth, maintaining short-term memory, identifying one's own name, discerning the current season, and recognizing one's location. Individuals were also regarded as having a cognitive disorder if they sometimes or always experienced wandering or difficulty returning home.

We classified an individual as possessing a mental or behavioral disorder if he or she demonstrated any of the following symptoms, either sometimes or always: paranoid behavior, confabulation, emotional instability, disrupted circadian rhythm, repetitive speech, elevated vocal volume, resistance to care, restlessness coupled with the desire to return home, tendency to venture out of the home alone, hoarding disorder, destructive behavior towards objects or clothing, severe forgetfulness, purposeless talking or laughter, inappropriate self-centered behavior, or incoherent speech. An individual who underwent any of the following medical treatments within 2 weeks prior to the certification survey date was categorized as having received medical treatment: medical infusion, central venous nutrition, dialysis, stoma management, oxygen therapy, ventilator utilization, tracheotomy care, pain management, enteral nutrition, monitoring measurements (blood pressure, heart rate, oxygen saturation, or other parameters), treatment of pressure ulcers, and catheterization.

### Statistical analyses

We first matched the exposed group (users) to the control group (non-users) by using propensity scores, which were estimated using the sex, age group, support/care-need level(s), paralysis, contractures, cognitive disorder, mental or behavioral disorder, and medical care. After the estimation, 1:1 matching without replacement was done using the 5 → 1 digit greedy algorithm [19], which is computationally efficient. The matching was done at the level of each insurer (i.e., local government) to control for area-level variables, including the area-level socioeconomic status of residents, the degree of urbanization, and the surrounding environment.

We assessed the balances of covariates by determining the standardized mean difference [20]. We considered a standardized mean difference of > 0.1 as an imbalance. Using the certification data up until October 2017, we calculated the follow-up time for each individual (in months as the unit) from 6 months after the first certification to the month in which progression in a support/care-need level was observed, or to the latest renewed-certification month. Individuals who moved out of the local government's area during the follow-up were censored before the transfer.

Kaplan–Meier curves analyses and log-rank tests were applied to compare the increase in support- and care-need levels between the exposed and control groups. As the proportional hazard assumptions were not satisfied, Cox proportional hazard models were not applicable. We performed stratification by individual characteristics of age groups, sex, and initial support/care-need levels. Within each subgroup, we repeated the propensity score calculation and 1:1 matching. We also performed stratification analyses of regional characteristics based on urban–rural classifications (urban, intermediate, and rural) and regions (Hokkaido, Tohoku, Kanto, Chubu, Kinki, Chugoku/Shikoku, Kyushu/Okinawa). Since matching in the primary analysis was conducted at the level of the insurer (i.e., local government), rematching by the recalculated propensity score was unnecessary for the stratification analysis by regional characteristics. We used the population density provided the individual's insurer (i.e., local municipality) as a proxy for the urban–rural classification [21] and classified the population density into three categories: rural (first tertile), intermediate (second tertile), and urban (third tertile). The cutoff values of population density were 476.8/km^2^ and 2,415.1/km^2^, respectively. We followed a previous study for the regional classification [22]. Statistical analyses were conducted using R (ver. 4.1.1) and Python 3 (ver. 3.8.10).

## Results

**Fig. 1:**
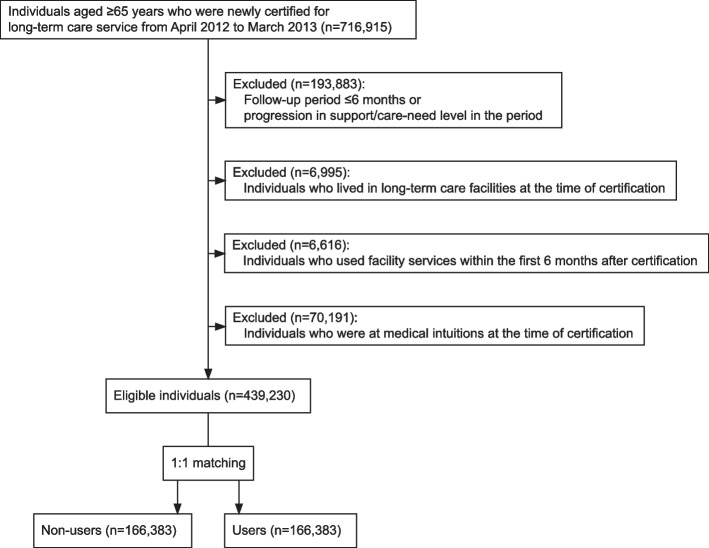
Subject selection chart

We initially identified 439,230 eligible individuals from 1,722 insurers in the study period (Fig. 1). Prior to matching, 197,769 (45.0%) were categorized as non-users, and 241,461 (55.0%) were identified as users. After the 1:1 propensity score matching, 332,766 individuals remained for the subsequent analyses. The mean follow-up period of these individuals was 22.4 months. The characteristics of this study populations before and after the propensity score matching are summarized in Table 1. After matching, the characteristics were balanced between the two groups.

**Table 1:** Demographic characteristics before and after matching of the users and non-users of Japan's long-term care system

	**Before propensity score matching**			**After propensity score matching**		
	**Non-users**	**Users**	**SMD**	**Non-users**	**Users**	**SMD**
n	197,769	241,461		166,383	166,383	
Age, yrs, n (%):			0.077			0.019
< 75	35,669 (18.0)	42,070 (17.4)		29,808 (17.9)	29,987 (18.0)	
75–79	46,606 (23.6)	51,205 (21.2)		38,374 (23.1)	37,091 (22.3)	
80–84	60,559 (30.6)	73,756 (30.5)		50,723 (30.5)	51,133 (30.7)	
≥ 85	54,935 (27.8)	74,430 (30.8)		47,478 (28.5)	48,172 (29.0)	
Sex, n (%):			0.050			0.037
Female	128,755 (65.1)	162,954 (67.5)		108,982 (65.5)	111,902 (67.3)	
Male	69,014 (34.9)	78,507 (32.5)		57,401 (34.5)	54,481 (32.7)	
Support/Care-need level, n (%):			0.312			0.010
Support-need level 1	94,032 (47.5)	83,987 (34.8)		73,013 (43.9)	72,288 (43.4)	
Support-need level 2	55,498 (28.1)	67,022 (27.8)		48,394 (29.1)	49,098 (29.5)	
Care-need level 1	48,239 (24.4)	90,452 (37.5)		44,976 (27.0)	44,997 (27.0)	
Paralysis, n (%):			0.040			< 0.001
Yes	58,437 (29.5)	75,771 (31.4)		49,661 (29.8)	49,669 (29.9)	
No	139,332 (70.5)	165,690 (68.6)		116,722 (70.2)	116,714 (70.1)	
Contractures, n (%):			0.020			0.011
Yes	39,757 (20.1)	50,468 (20.9)		34,036 (20.5)	33,282 (20.0)	
No	158,012 (79.9)	190,993 (79.1)		132,347 (79.5)	133,101 (80.0)	
Cognitive disorder, n (%):			0.163			0.001
Yes	37,679 (19.1)	62,337 (25.8)		34,185 (20.5)	34,105 (20.5)	
No	160,090 (80.9)	179,124 (74.2)		132,198 (79.5)	132,278 (79.5)	
Mental or behavioral disorder, n (%):			0.172			0.009
Yes	75,320 (38.1)	112,410 (46.6)		66,806 (40.2)	67,544 (40.6)	
No	122,449 (61.9)	129,051 (53.4)		99,577 (59.8)	98,839 (59.4)	
Medical care received, n (%):			0.055			0.009
Yes	9,785 ( 4.9)	9,246 ( 3.8)		7,025 ( 4.2)	6,730 ( 4.0)	
No	187,984 (95.1)	232,215 (96.2)		159,358 (95.8)	159,653 (96.0)	
Urban–rural classification, n (%):			0.167			—
Urban	75,358 (38.1)	73,991 (30.6)		58,352 (35.1)	58,352 (35.1)	
Intermediate	62,976 (31.8)	80,510 (33.3)		53,976 (32.4)	53,976 (32.4)	
Rural	59,435 (30.1)	86,960 (36.0)		54,055 (32.5)	54,055 (32.5)	
Region, n (%):			0.195			—
Hokkaido	10,538 ( 5.3)	12,625 ( 5.2)		9,489 ( 5.7)	9,489 ( 5.7)	
Tohoku	13,592 ( 6.9)	19,870 ( 8.2)		12,519 ( 7.5)	12,519 ( 7.5)	
Kanto	54,061 (27.3)	62,465 (25.9)		47,370 (28.5)	47,370 (28.5)	
Chubu	29,458 (14.9)	48,330 (20.0)		28,154 (16.9)	28,154 (16.9)	
Kinki	47,261 (23.9)	43,018 (17.8)		33,419 (20.1)	33,419 (20.1)	
Chugoku/Shikoku	21,935 (11.1)	30,087 (12.5)		20,167 (12.1)	20,167 (12.1)	
Kyushu/Okinawa	20,924 (10.6)	25,066 (10.4)		15,265 ( 9.2)	15,265 ( 9.2)	

*SMD* standardized mean difference

**Fig. 2:**
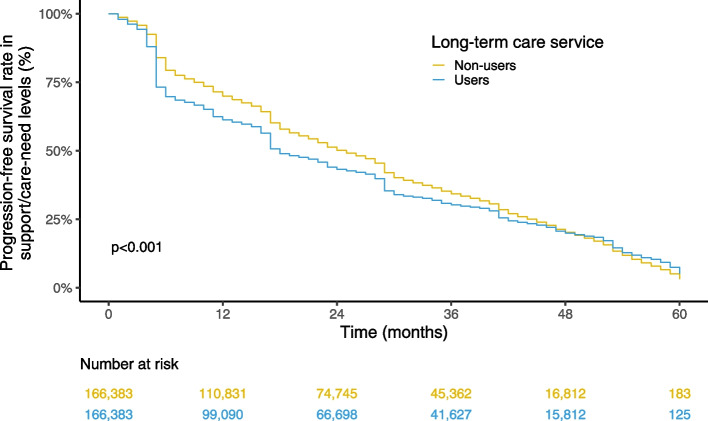
Kaplan–Meier curve estimates of support/care-need level progression in the whole study population

Overall, the 6-, 12-, 36-, and 60-month progression-free survival estimates in the non-users of the long-term care system were 79.4% (79.2%–79.6%), 70.0% (69.8%–70.2%), 34.3% (34.0%–34.5%), and 3.2% (2.8%–3.6%), respectively (Fig. 2). For the users of the long-term care system, the corresponding estimates were 69.7% (69.5%–69.9%), 61.3% (61.1%–61.5%), 30.2% (30.0%–30.4%), and 5.2% (4.6%–5.9%), respectively. The differences in these data between the users and non-users were significant (log-rank test, *p* < 0.001).

**Fig. 3:**
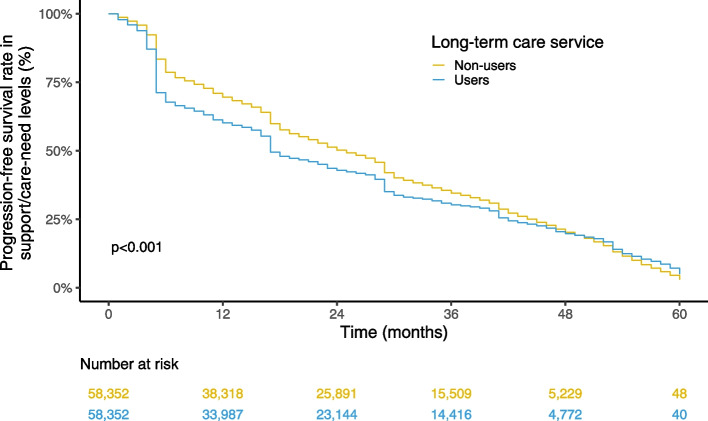
Kaplan–Meier curve estimates of support/care-need level progression in the urban areas

**Fig. 4:**
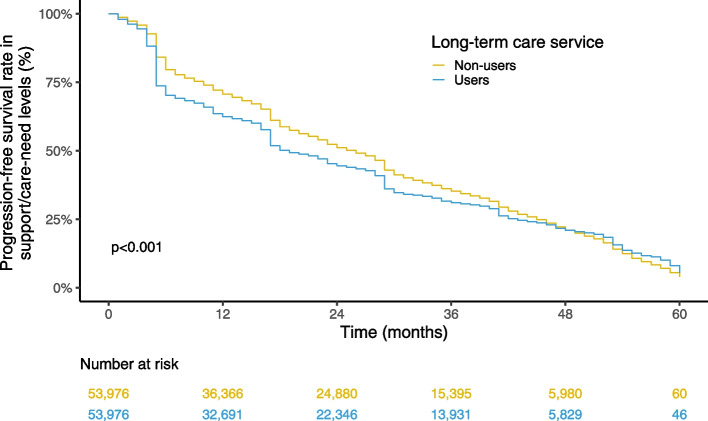
Kaplan–Meier curve estimates of support/care-need level progression in the intermediate urban/rural areas

**Fig. 5:**
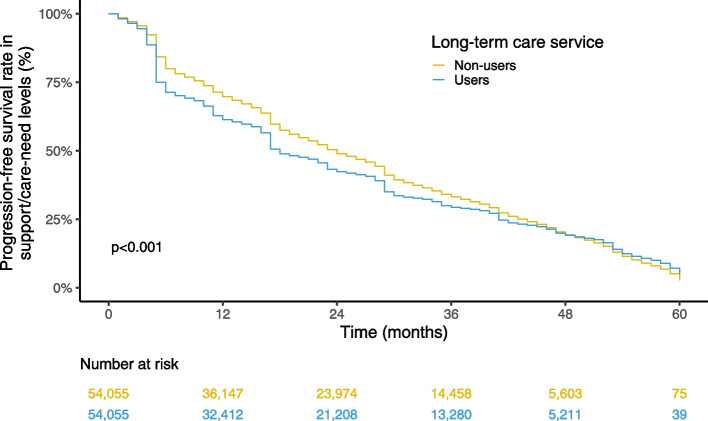
Kaplan–Meier curve estimates of support/care-need level progression in the rural areas

Supplementary Tables S1–S9 summarizes the subjects' characteristics before and after matching for the stratification analysis by age groups, sex, and initial support/care-need levels. After the matching, the characteristics were balanced between the users and non-users in each stratum. The results of each stratum were similar to those observed in the overall analysis (Suppl. Figs. S1–S9). When stratified for urban–rural classifications, the results were almost the same as those of the primary analysis in all three urban–rural classifications (Figs. 3, 4 and 5). The results were also nearly unchanged when the data were stratified by the seven geographic regions, although the log-rank test showed nonsignificance for the Kyushu-Okinawa region (Suppl. Figs. S10–S16).

## Discussion

This retrospective cohort study examined the association between the use of Japan's long-term care service and the progression in support- and care-need levels at the national scale. The results of our analyses demonstrated that using the long-term care service was associated with greater progression (i.e., a faster decline) in support/care-need levels, although the differences in the subjects' survival rate subsequently diminished. The results were essentially the same in our stratification analyses by age groups, sex, initial support/care-need levels, urban–rural classification, and region.

Earlier studies of long-term care service use in Japan obtained conflicting results [3–7]. For example, a study of 3,006 elderly persons in a Tokyo ward reported that any service use prevented the progression of care-need levels [3]. An investigation of 2,651 individuals in the city of Izumo reported that home help and bathing service use were associated with sustaining care-need levels, but daycare service and short-stay service were associated with a deterioration in care-need levels [5]. A recent study of 1,289 persons in the city of Kashiwa indicated that service usage suppressed the deterioration in the level of care needed in a subgroup of individuals aged ≥ 85 and at support-need level 1, but not in other subgroups [4]. The inconsistent outcomes of these studies may be attributed to variations such as differences in the study population, study period, and statistical methods employed. The relatively modest sample sizes may also have contributed to the observed inconsistency.

Our present analyses were at the national scale, and we observed faster declines in survival curves among the users of the long-term care system, although the difference was subsequently diminished. One possible explanation for the decline in survival among users is the uncontrolled differences in characteristics between the user and non-user groups [5, 6]. It is also possible that the service users had poorer health statuses compared to the non-users, although our statistical analyses controlled for some comorbidity statuses that were available in the database. Another possible explanation is that there may have been an incentive for service users to under-report their health status in the certification survey, as recipients can receive more service when they are certified for higher support/care-need levels. Although declines in the support- or care-need level have been examined in several studies [3–6, 10, 17, 18], this measure may be inappropriate for assessing the progression of disability.

However, combined with previous results, our present findings indicate that Japan's long-term care services might not have the expected beneficial effects on the recipients of services. Similarly, the effects of preventive healthcare programs on elderly populations have been questioned globally. A systematic review of home visiting programs' effects on elderly individuals concluded that home visits are not associated with the prevention of negative health outcomes [23]. A recent trial reported that targeted interventions for older people did not reduce the risk of falls [24]. As the total costs for Japan's long-term care system are rapidly increasing [2], a re-examination of this system toward the goal of providing more cost-effective services may be worthwhile.

Our present findings demonstrated that approx. 45.0% of the newly certified individuals identified did not use any service during the initial 6 months after their certification. Comparable proportions of non-users among certified individuals have been documented in other investigations: e.g., 16.7% [3], 55.2% [4], and 39.0% [25]. We were unable to determine the reasons for the non-utilization of long-term care services among certified individuals from the database used in the present study. Nonetheless, another investigation reported that 40.4% of the non-users applied for certification for future service utilization [25]. Such characteristics among non-users may have partly contributed to the slower decline in support/care-need levels revealed by our present analyses.

By using a nationwide study design herein, we explored regional variations in the effects of service use; these variations were not explored previously. We observed that the ratio of the system's users among the newly certified individuals differed significantly by urban–rural classifications or regions, as has been reported [26]. However, no clear variations in service use were revealed by these stratification analyses. These results imply that there might be no clear benefits of using Japan's long-term care service, even in areas where excessive service use was most unlikely [27], and they further support the possibility that the long-term care system has been ineffective.

There are several study limitations to consider. First, we could not adjust for several confounders which could have affected our estimates. Although we controlled the area-level socioeconomic status data by performing matching at the level of each insurer (i.e., the local municipality), our analyses did not include individual-level socioeconomic status information such as household income and education. We also considered several individual-level comorbidity statuses available in the database, but other comorbidities were not considered, as linking with other data sources was prohibited when this study was planned. However, the MHLW has since started linking the Japanese Long-Term Care Insurance Claims database and other databases, including a nationwide claims database. In addition, some researchers have developed databases that integrate multiple health databases, including long-term care insurance claims and medical claims [28, 29]. Future studies using such data are warranted.

Second, we defined use of the long-term care system by applying only the long-term care insurance claim data during a 6-month period after the individuals' initial certification, and the service use after that period was not examined. In addition, individuals who used any service at least once were grouped as users, and the service intensity and the types of service were not considered. There could also be differences in the effects of the service among the many service providers across the country. We used the current definition of exposure of interest for clarity and methodological convenience, but future studies should consider the heterogeneity of services.

Third, we used changes in support/care-need levels as a proxy for the subjects' health status, and we could not capture effects on other adverse health outcomes such as mortality, hospitalizations, and medical costs [30]. Although we were unable to examine effects on the support/care-need level changes, there could have been beneficial effects on other health outcomes. In addition, the impact of use/non-use of the long-term care system on caregivers was not included in this study, but previous investigations indicated that some services could improve the health status of caregivers [31, 32]. Fourth, our results based on urbanization may be interpreted differently, since we used population density as a proxy for the degree of urbanization. Finally, although this was a nationwide study that covered nearly all of Japan, the applicability of our results to other countries is likely to be limited.

## Conclusion

This nationwide study revealed that the use of long-term care services in Japan was not associated with preventing deterioration in the recipients' support-need or care-need levels. Our findings indicate a need to reconsider the system from the viewpoint of the sustainability of its services.

## Supplementary Information

**Additional file 1: Suppl. Fig. S1.** Kaplan-Meier curve estimates of support/care-need level progression among individuals aged <75 years.** Suppl. Fig. S2.** Kaplan-Meier curve estimates of support/care-need level progression among individuals aged 75–79 years.** Suppl. Fig. S3.** Kaplan-Meier curve estimates of support/care-need level progression among individuals aged 80–84 years.** Suppl. Fig. S4.** Kaplan-Meier curve estimates of support/care-need level progression among individuals aged ≥85 years.** Suppl. Fig. S5.** Kaplan-Meier curve estimates of support/care-need level progression among the females.** Suppl. Fig. S6.** Kaplan-Meier curve estimates of support/care-need level progression among the males.** Suppl. Fig. S7.** Kaplan-Meier curve estimates of support/care-need level progression among individuals with support-need level 1 at baseline.** Suppl. Fig. S8.** Kaplan-Meier curve estimates of support/care-need level progression among individuals with support-need level 2 at baseline.** Suppl. Fig. S9.** Kaplan-Meier curve estimates of support/care-need level progression among individuals with care-need level 1 at baseline.** Suppl. Fig. S10.** Kaplan-Meier curve estimates of support/care-need level progression in the Hokkaido region.** Suppl. Fig. S11.** Kaplan-Meier curve estimates of support/care-need level progression in the Tohoku region.** Suppl. Fig. S12.** Kaplan-Meier curve estimates of support/care-need level progression in the Kanto region.** Suppl. Fig. S13.** Kaplan-Meier curve estimates of support/care-need level progression in the Chubu region.** Suppl. Fig. S14.** Kaplan-Meier curve estimates of support/care-need level progression in the Kinki region.** Suppl. Fig. S15.** Kaplan-Meier curve estimates of support/care-need level progression in the Chugoku/Shikoku region.** Suppl. Fig. S16.** Kaplan-Meier curve estimates of support/care-need level progression in the Kyushu/Okinawa region.** Suppl. Table S1.** Demographic characteristics of the individuals aged <75 before and after the matching of the users and non-users of Japan's long-term care system.** Suppl. Table S2.** Demographic characteristics of the individuals aged 75–79 before and after the matching of the users and non-users of Japan's long-term care system.** Suppl. Table S3.** Demographic characteristics of the individuals aged 80–84 before and after the matching of the users and non-users of Japan's long-term care system.** Suppl. Table S4.** Demographic characteristics of the individuals aged ≥85 before and after the matching of the users and non-users of Japan's long-term care system.** Suppl. Table S5.** Demographic characteristics of the females before and after the matching of the users and non-users of Japan's long-term care system.** Suppl. Table S6.** Demographic characteristics of the males before and after the matching of the users and non-users of Japan's long-term care system.** Suppl. Table S7.** Demographic characteristics of the individuals with support-need level 1 at baseline before and after the matching of the users and non-users of Japan's long-term care system.** Suppl. Table S8.** Demographic characteristics of the individuals with support-need level 2 at baseline before and after the matching of the users and non-users of Japan's long-term care system.** Suppl. Table S9.** Demographic characteristics of the individuals with care-need level 1 at baseline before and after the matching of the users and non-users of Japan's long-term care system.

## Data Availability

Our permission to use the Japanese Long-Term Care Insurance Claims database expired after the authorized research period, and we can no longer access the data. Therefore, anyone who want to access the data must apply to the MHLW. The codes used in the current study are available from the corresponding author upon request.

## Abbreviation

MHLW: Ministry of Health, Labour and Welfare
