# Supplementary material for: Associations between long-term care-service use and service- or care-need level progression: a nationwide cohort study using the Japanese Long-Term Care Insurance Claims database
Source: BMC Health Serv Res. 2023 Jun 5;23:577. doi: 10.1186/s12913-023-09615-0 (PMC10240700; doi:10.1186/s12913-023-09615-0)
Supplement: Supplementary file 1 — Additional file 1: Suppl. Fig. S1. Kaplan-Meier curve estimates of support/care-need level progression among individuals aged <75 years. Suppl. Fig. S2. Kaplan-Meier curve estimates of support/care-need level progression among individuals aged 75–79 years. Suppl. Fig. S3. Kaplan-Meier curve estimates of support/care-need level progression among individuals aged 80–84 years. Suppl. Fig. S4. Kaplan-Meier curve estimates of support/care-need level progression among individuals aged ≥85 years. Suppl. Fig. S5. Kaplan-Meier curve estimates of support/care-need level progression among the females. Suppl. Fig. S6. Kaplan-Meier curve estimates of support/care-need level progression among the males. Suppl. Fig. S7. Kaplan-Meier curve estimates of support/care-need level progression among individuals with support-need level 1 at baseline. Suppl. Fig. S8. Kaplan-Meier curve estimates of support/care-need level progression among individuals with support-need level 2 at baseline. Suppl. Fig. S9. Kaplan-Meier curve estimates of support/care-need level progression among individuals with care-need level 1 at baseline. Suppl. Fig. S10. Kaplan-Meier curve estimates of support/care-need level progression in the Hokkaido region. Suppl. Fig. S11. Kaplan-Meier curve estimates of support/care-need level progression in the Tohoku region. Suppl. Fig. S12. Kaplan-Meier curve estimates of support/care-need level progression in the Kanto region. Suppl. Fig. S13. Kaplan-Meier curve estimates of support/care-need level progression in the Chubu region. Suppl. Fig. S14. Kaplan-Meier curve estimates of support/care-need level progression in the Kinki region. Suppl. Fig. S15. Kaplan-Meier curve estimates of support/care-need level progression in the Chugoku/Shikoku region. Suppl. Fig. S16. Kaplan-Meier curve estimates of support/care-need level progression in the Kyushu/Okinawa region. Suppl. Table S1. Demographic characteristics of the individuals aged <75 before and after the matching of the users [file 12913_2023_9615_MOESM1_ESM.docx]

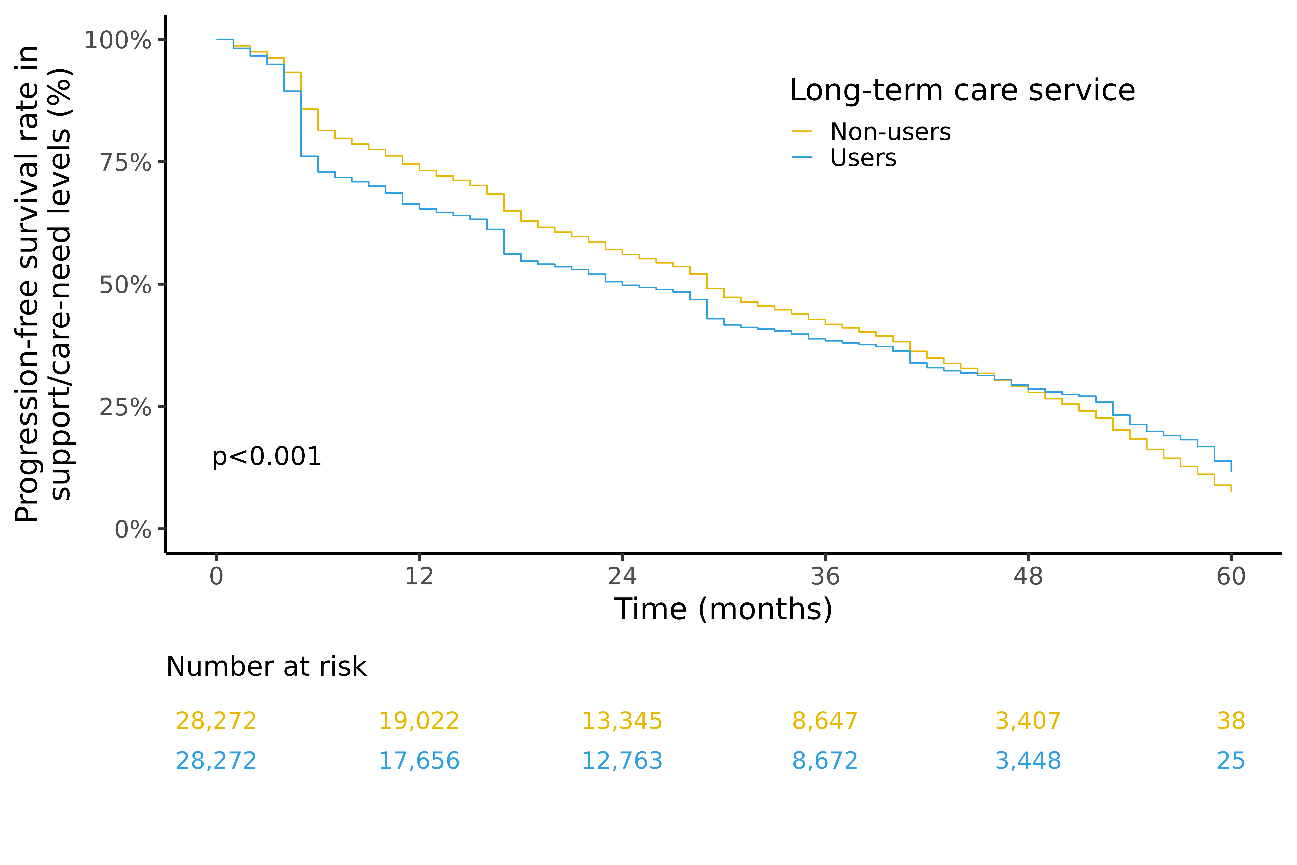


# Suppl. Fig. S1. Kaplan-Meier curve estimates of support/care-need level progression among individuals aged <75 years.


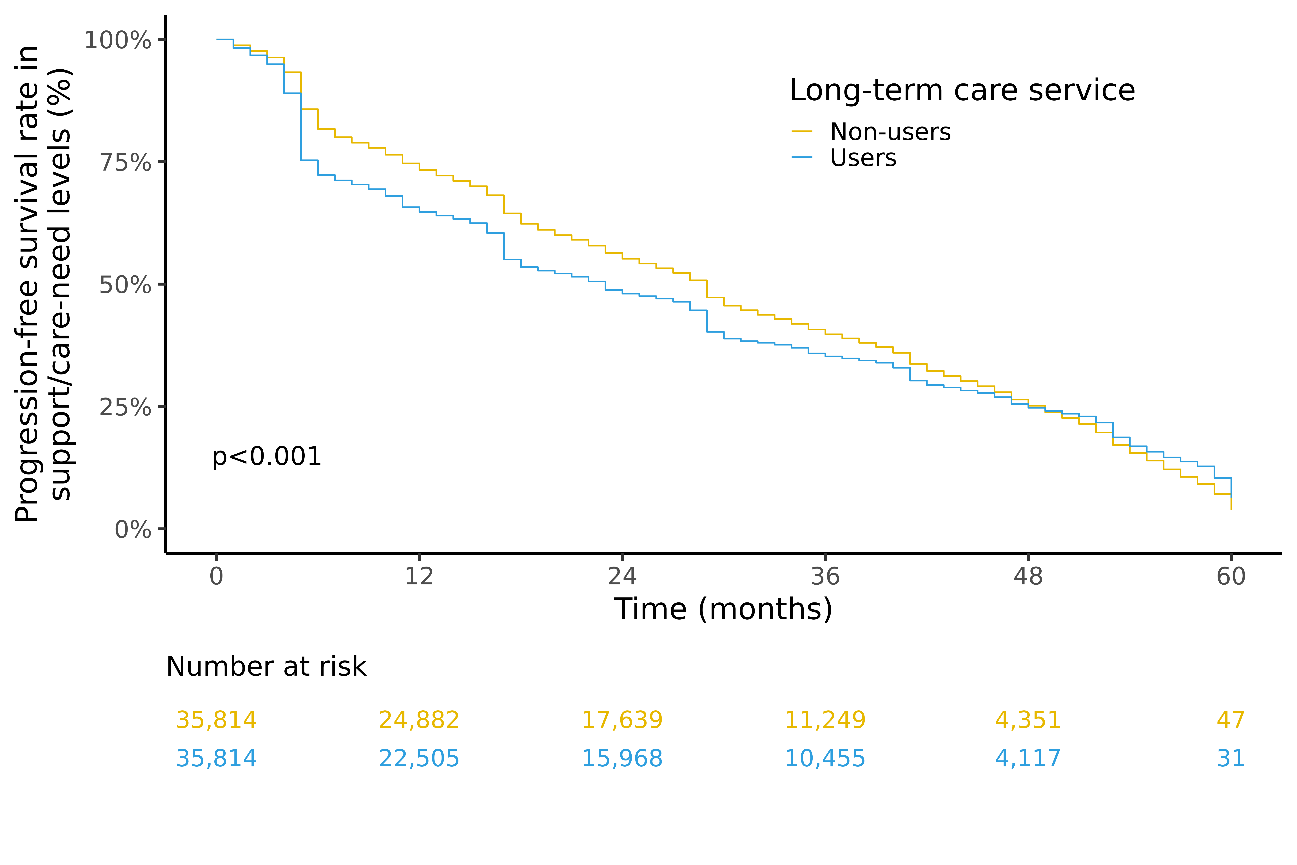


# Suppl. Fig. S2. Kaplan-Meier curve estimates of support/care-need level progression among individuals aged 75–79 years.


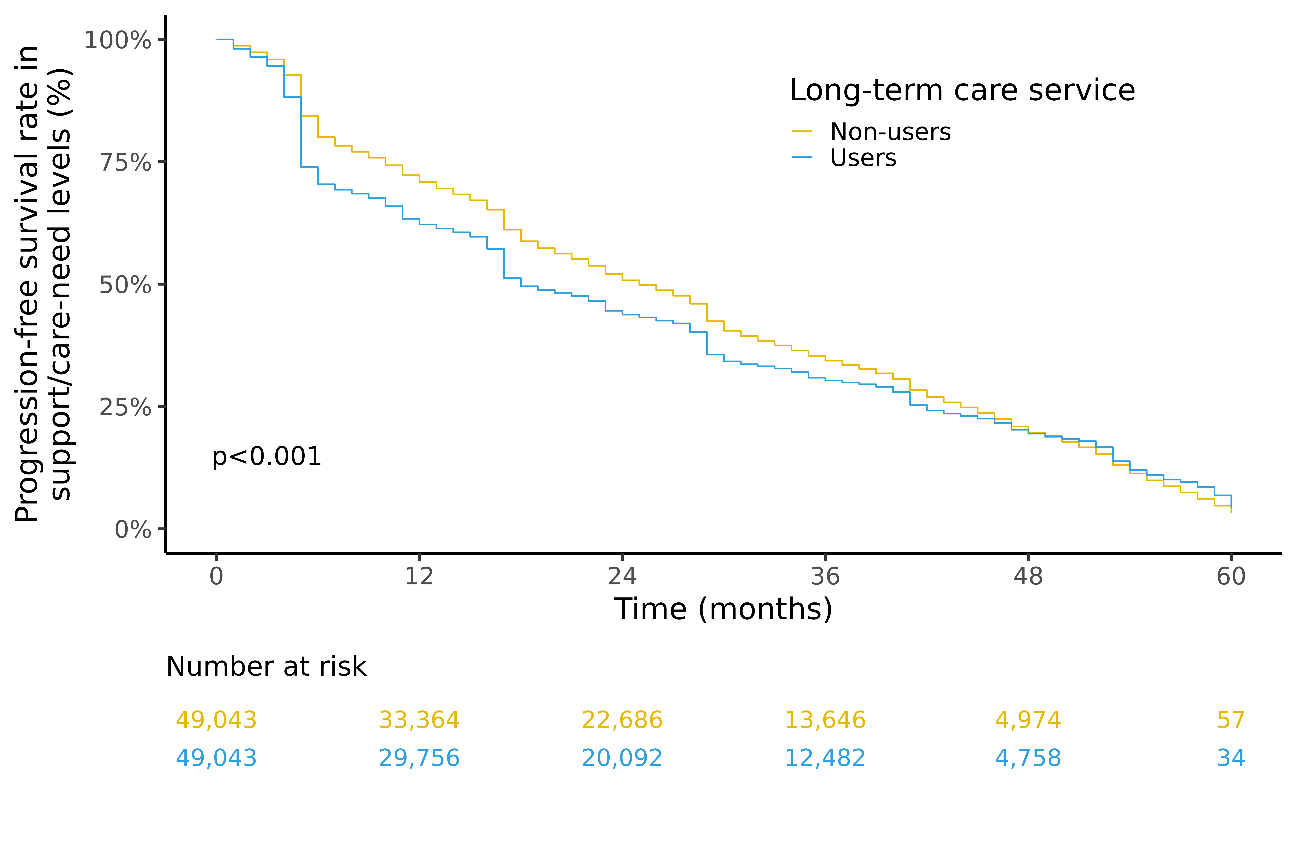


# Suppl. Fig. S3. Kaplan-Meier curve estimates of support/care-need level progression among individuals aged 80–84 years.


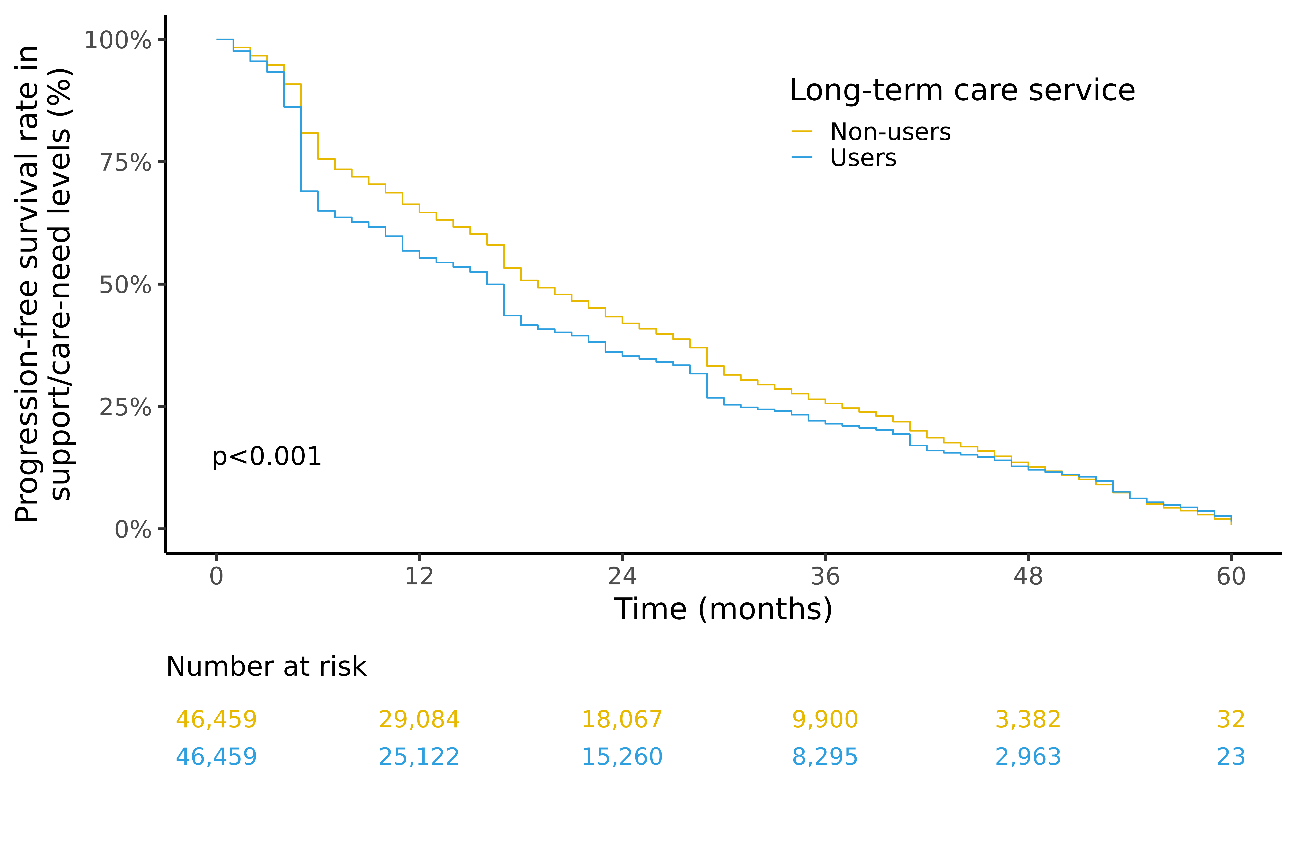


# Suppl. Fig. S4. Kaplan-Meier curve estimates of support/care-need level progression among individuals aged ≥85 years.


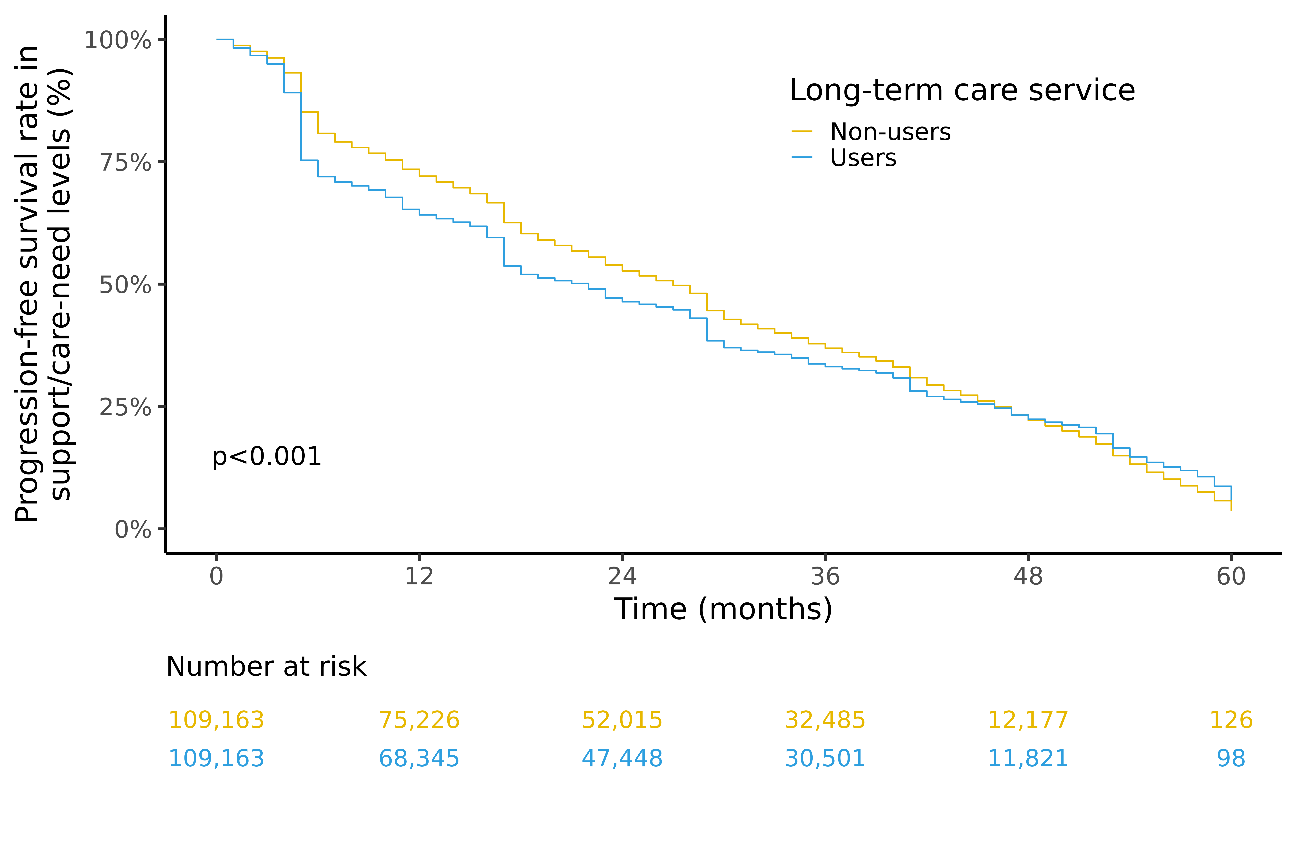


# Suppl. Fig. S5. Kaplan-Meier curve estimates of support/care-need level progression among the females.


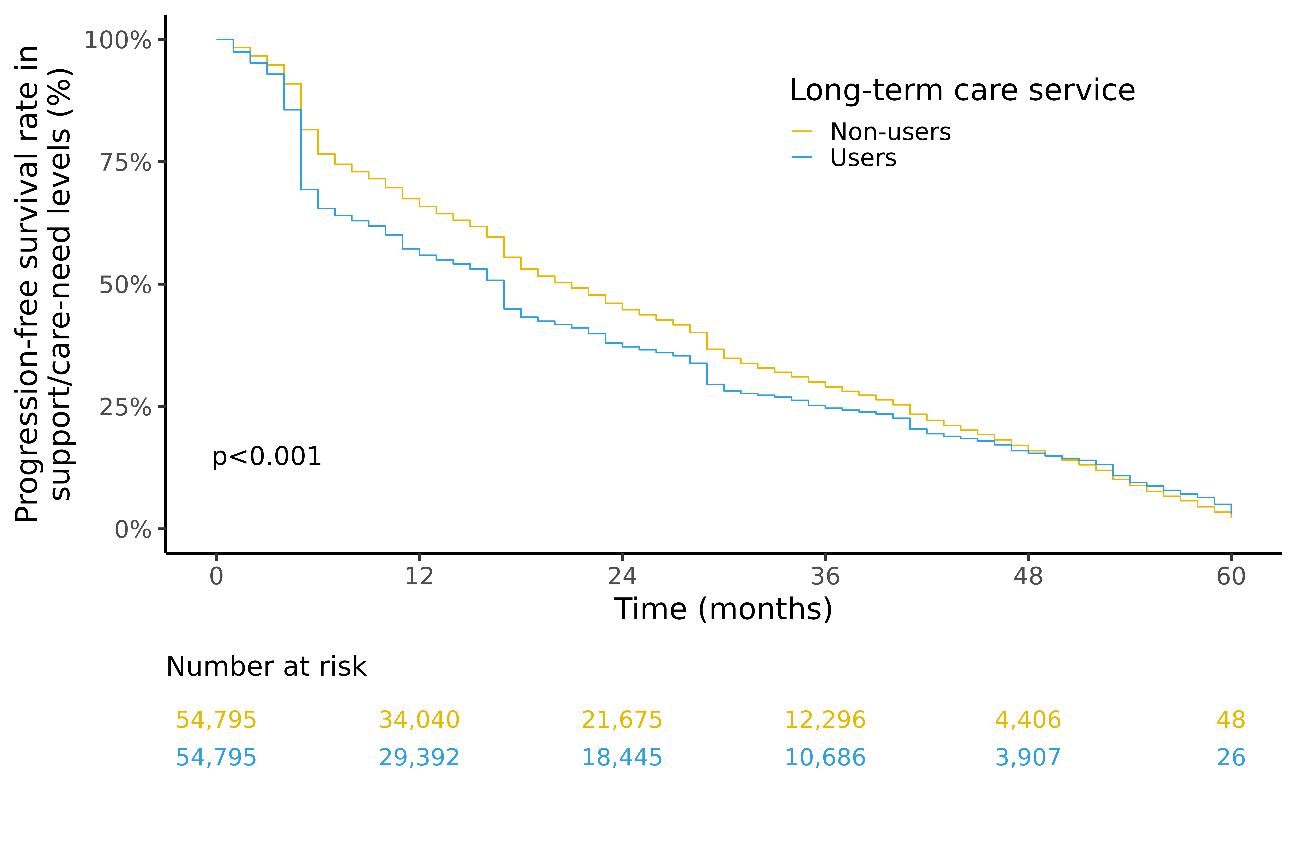


# Suppl. Fig. S6. Kaplan-Meier curve estimates of support/care-need level progression among the males.


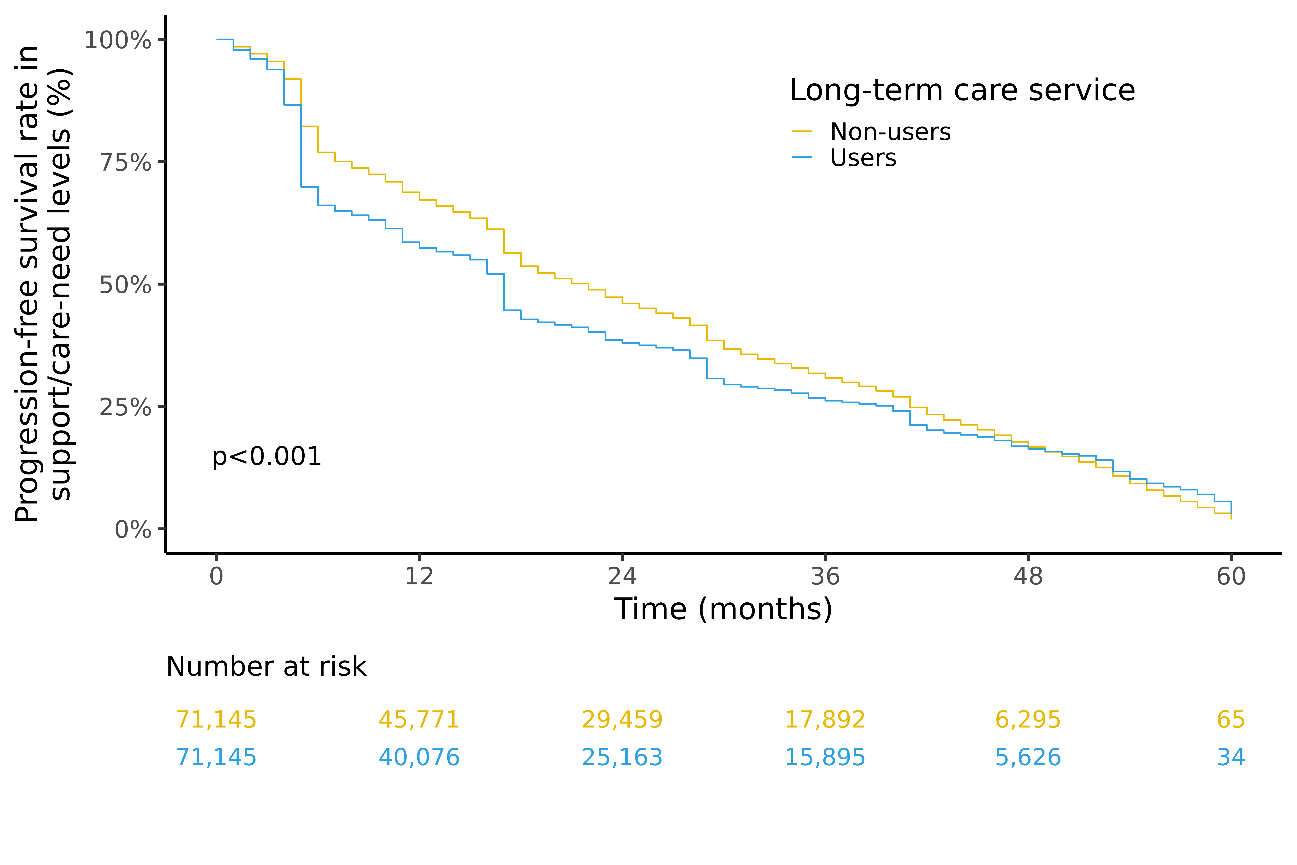


# Suppl. Fig. S7. Kaplan-Meier curve estimates of support/care-need level progression among individuals with support-need level 1 at baseline.


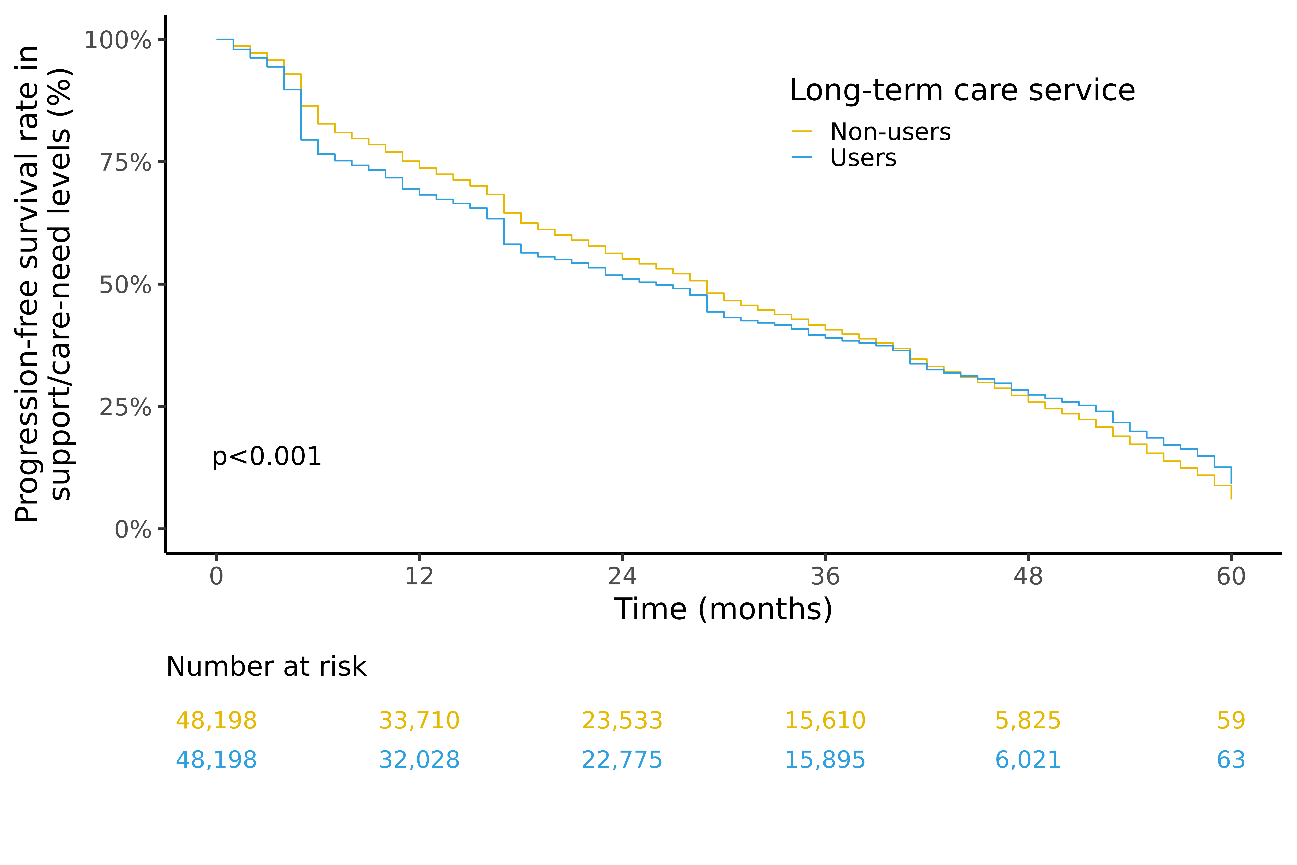


# Suppl. Fig. S8. Kaplan-Meier curve estimates of support/care-need level progression among individuals with support-need level 2 at baseline.


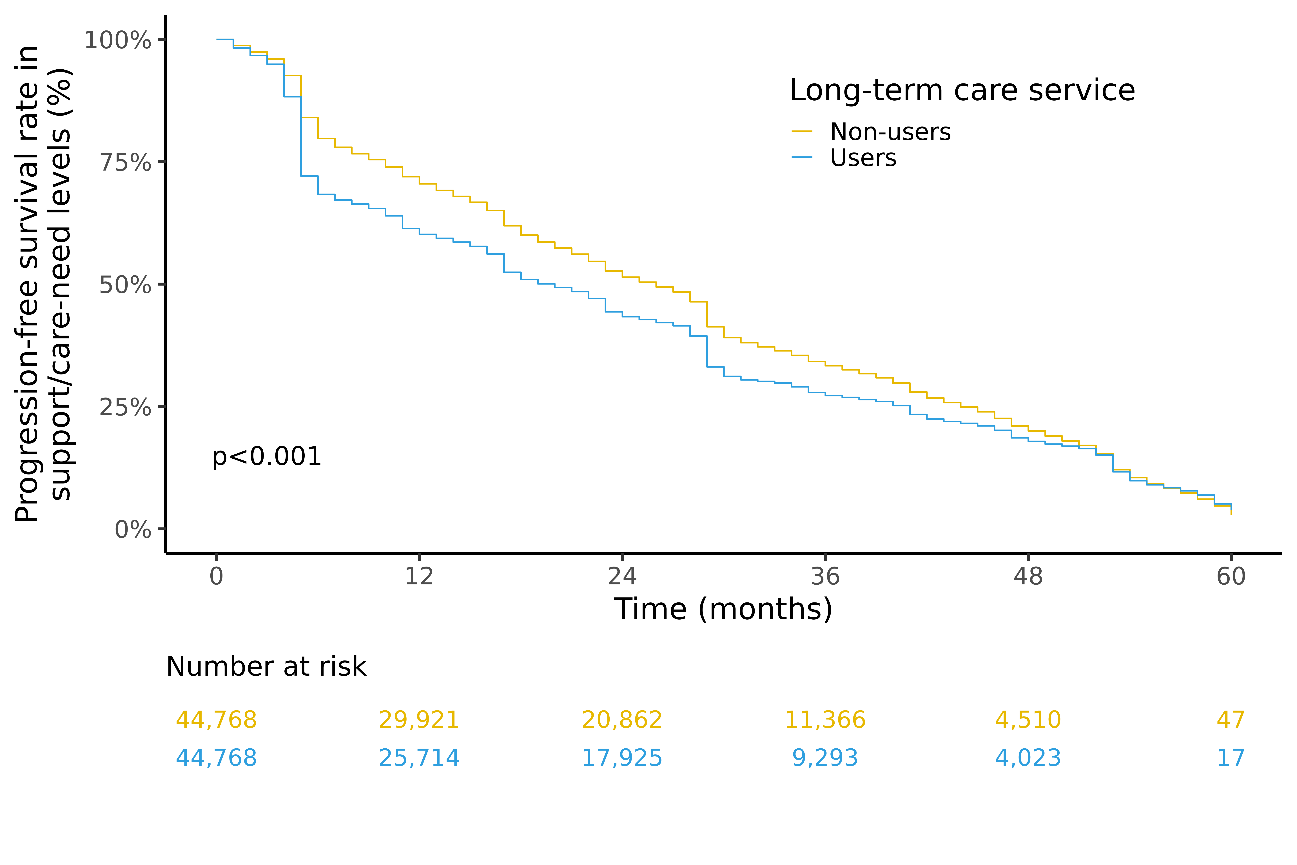


# Suppl. Fig. S9. Kaplan-Meier curve estimates of support/care-need level progression among individuals with care-need level 1 at baseline.


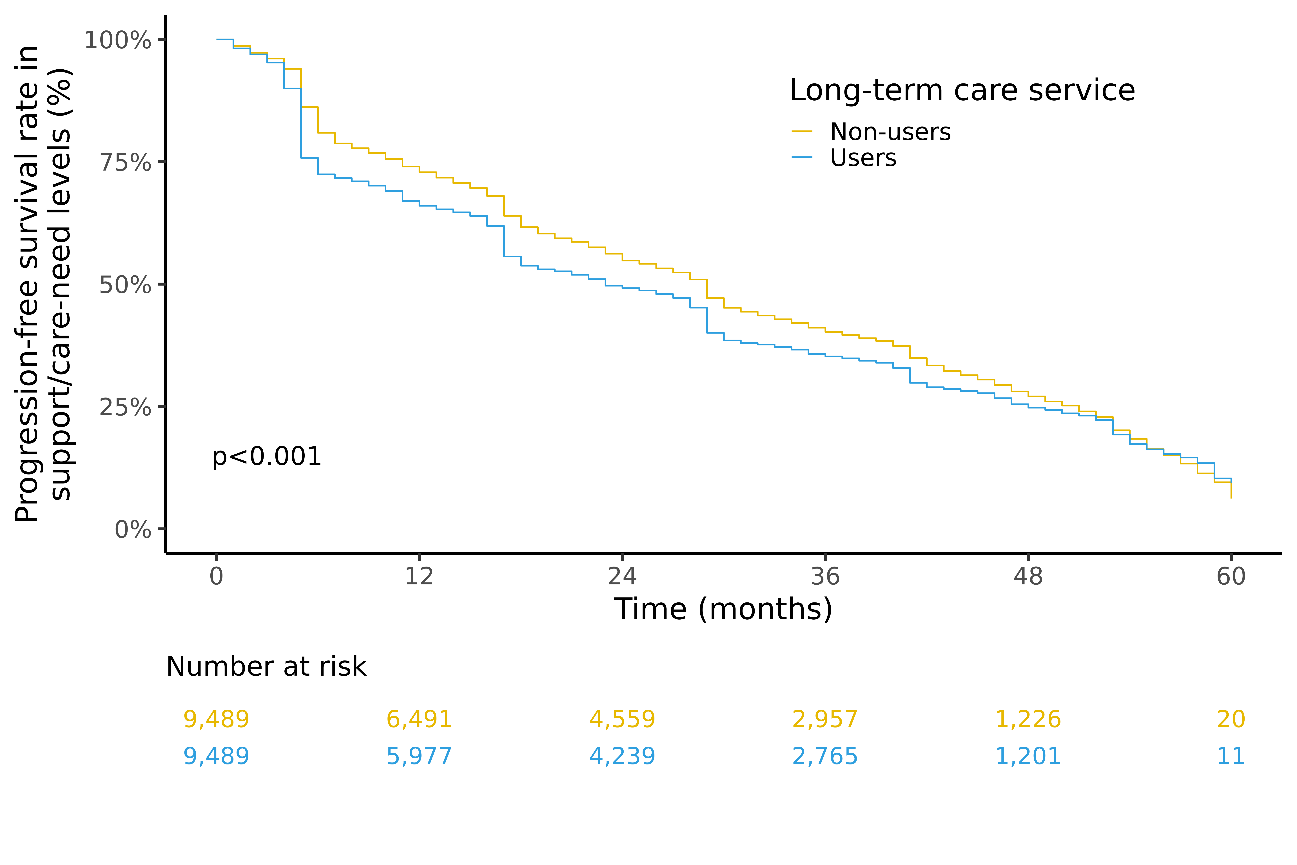


# Suppl. Fig. S10. Kaplan-Meier curve estimates of support/care-need level progression in the Hokkaido region.


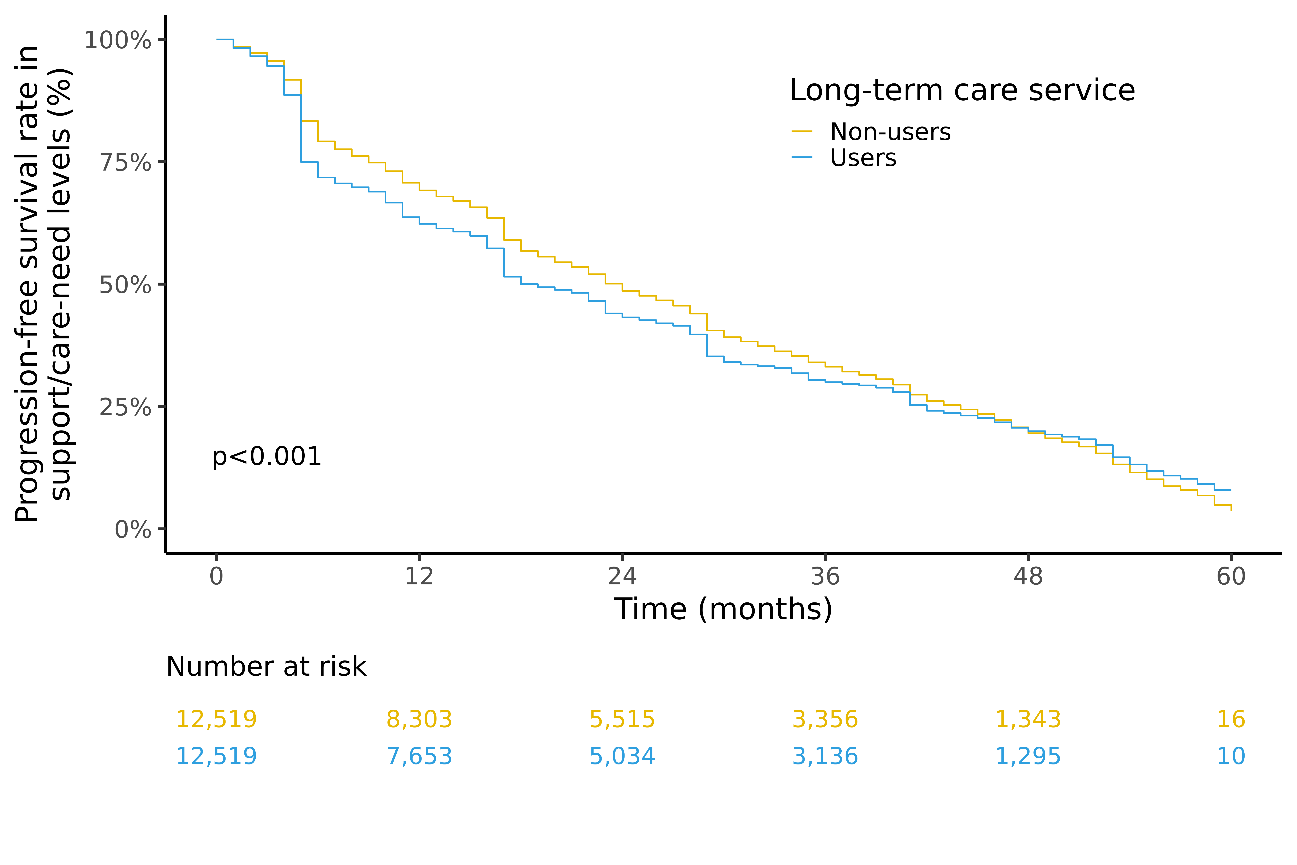


# Suppl. Fig. S11. Kaplan-Meier curve estimates of support/care-need level progression in the Tohoku region.


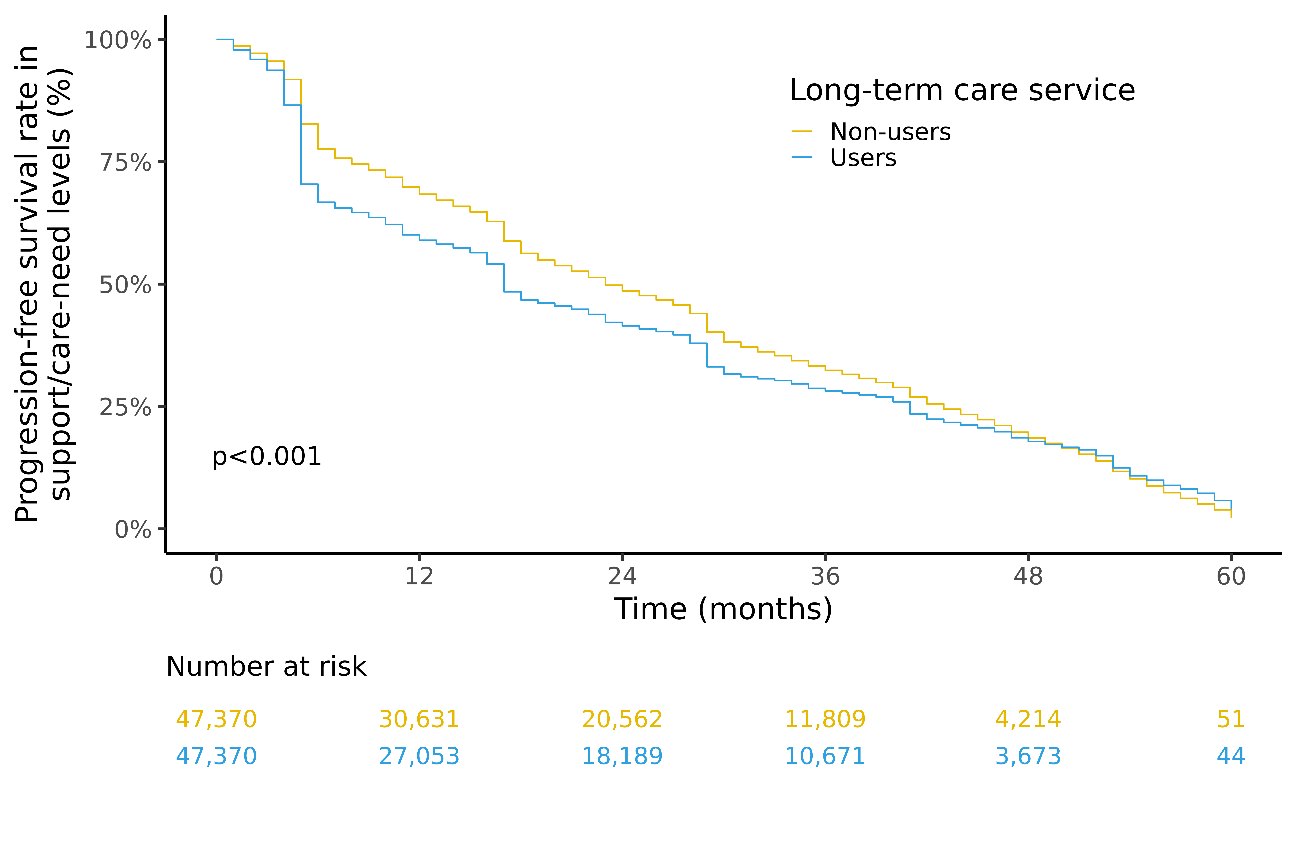


# Suppl. Fig. S12. Kaplan-Meier curve estimates of support/care-need level progression in the Kanto region.


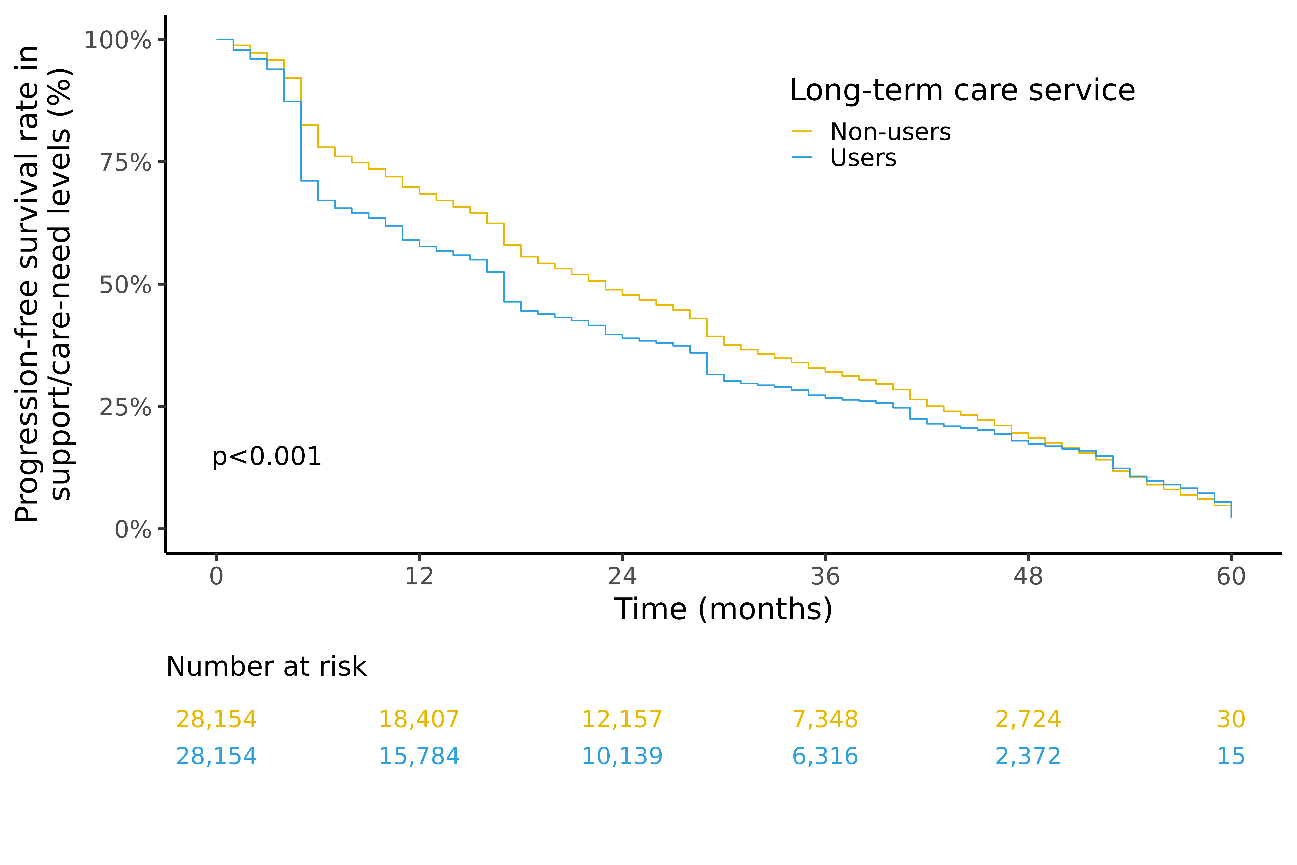


# Suppl. Fig. S13. Kaplan-Meier curve estimates of support/care-need level progression in the Chubu region.


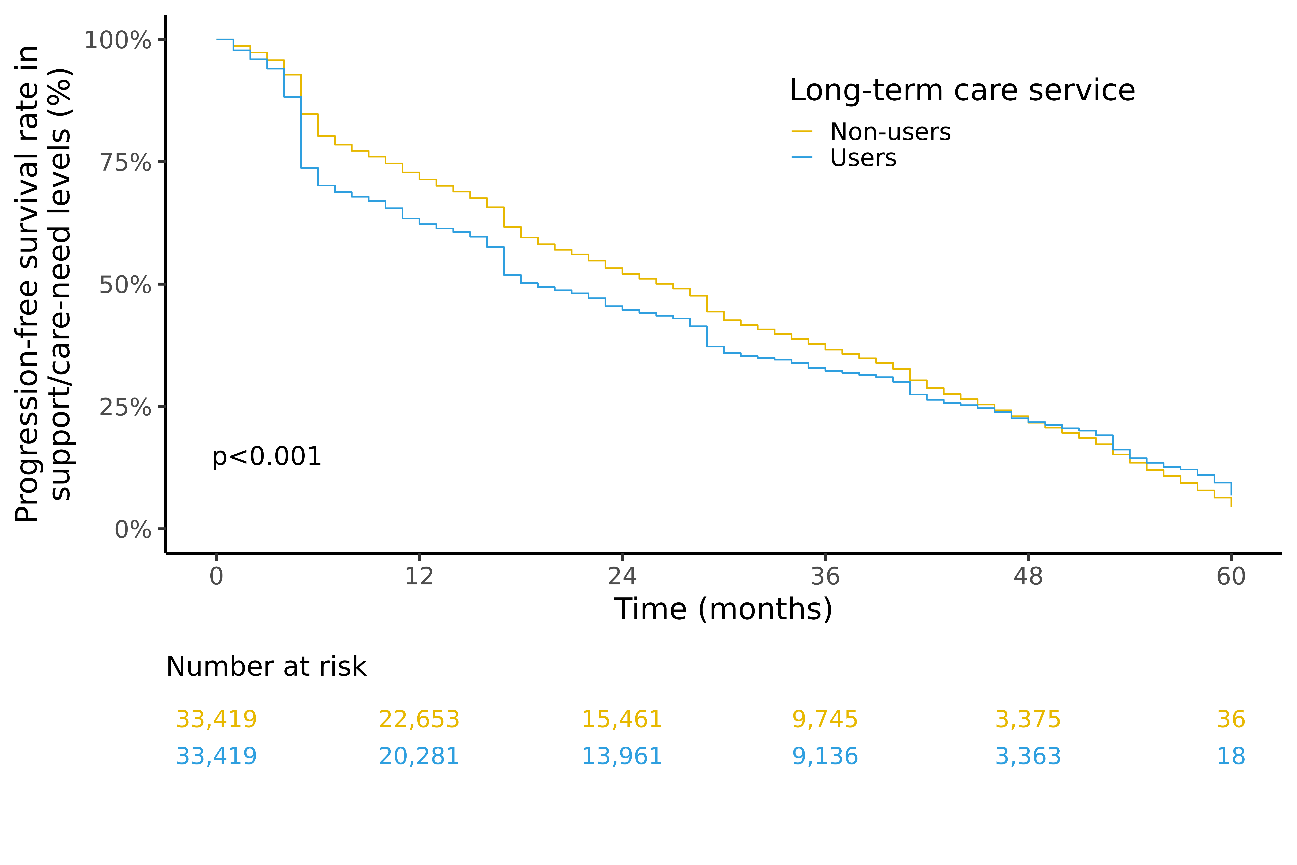


# Suppl. Fig. S14. Kaplan-Meier curve estimates of support/care-need level progression in the Kinki region.


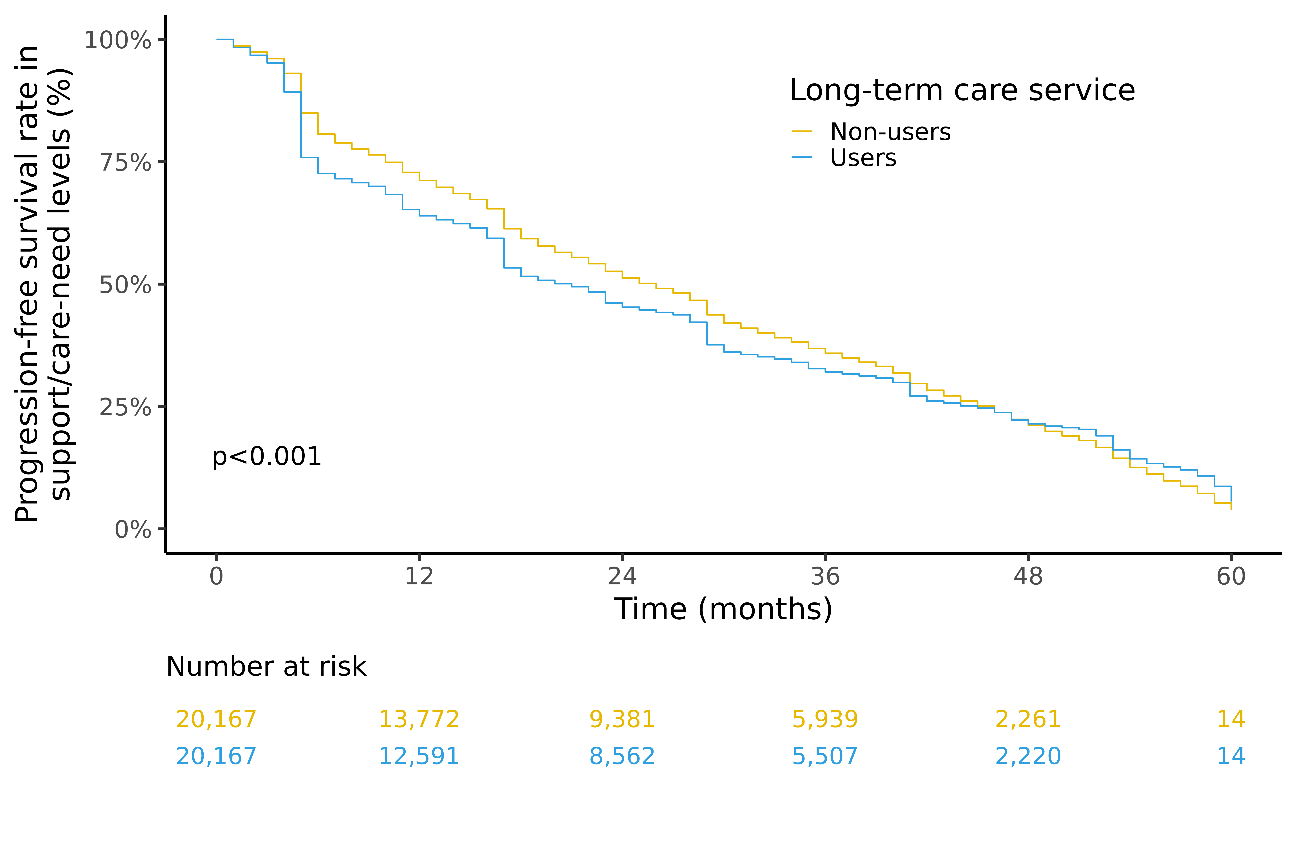


# Suppl. Fig. S15. Kaplan-Meier curve estimates of support/care-need level progression in the Chugoku/Shikoku region.


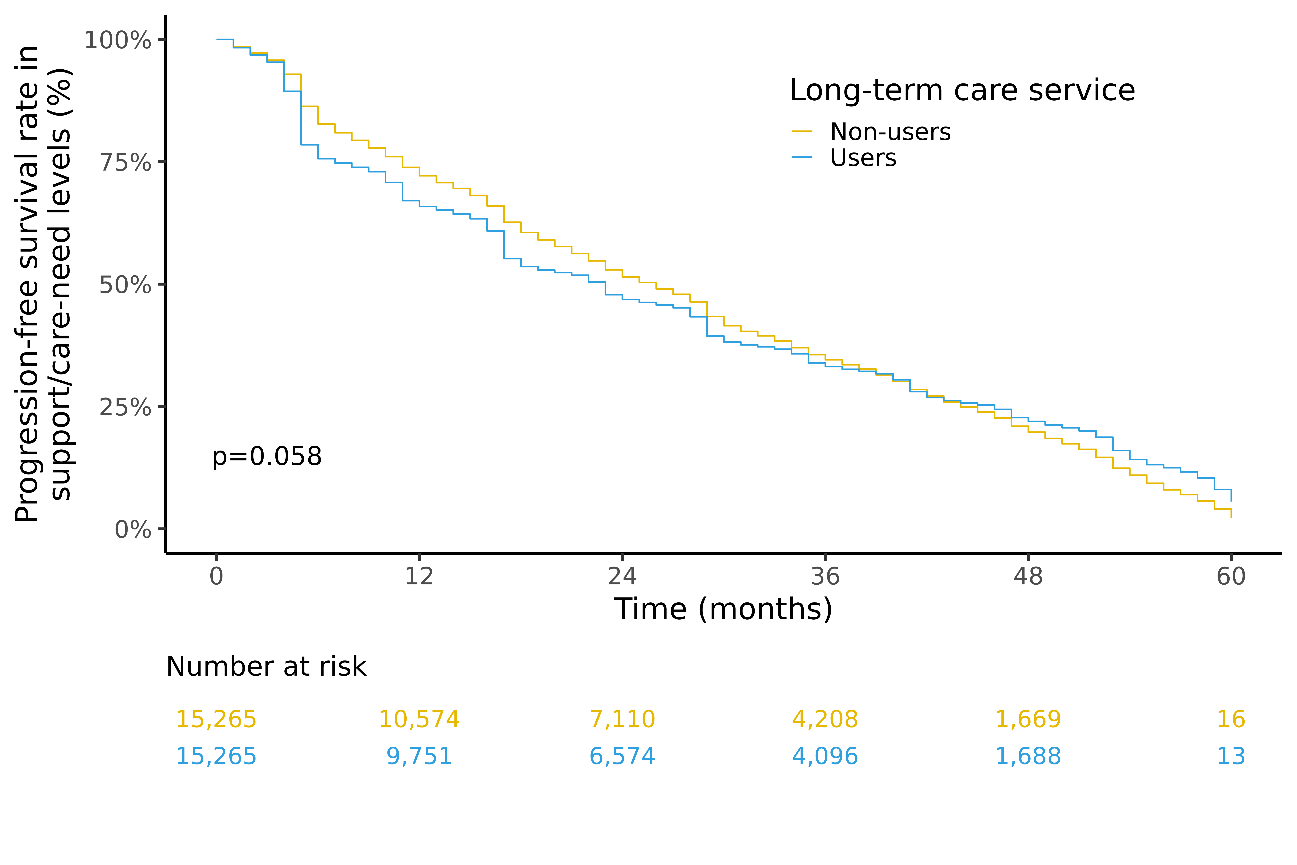


# Suppl. Fig. S16. Kaplan-Meier curve estimates of support/care-need level progression in the Kyushu/Okinawa region.

# Suppl. Table S1. Demographic characteristics of the individuals aged <75 before and after the matching of the users and non-users of Japan's long-term care system

|  | **Before propensity score matching** | | | **After propensity score matching** | | |
| --- | --- | --- | --- | --- | --- | --- |
|  | **Non-Users** | **Users** | **SMD** | **Non-users** | **Users** | **SMD** |
| n | 35,669 | 42,070 |  | 28,272 | 28,272 |  |
| Sex, n (%): |  |  | 0.068 |  |  | 0.017 |
| Female | 22,145.0 (62.1) | 24,716 (58.7) |  | 17,419.0 (61.6) | 17,180 (60.8) |  |
| Male | 13,524.0 (37.9) | 17,354 (41.3) |  | 10,853.0 (38.4) | 11,092 (39.2) |  |
| Support/Care-need level, n (%): |  |  | 0.275 |  |  | 0.015 |
| Support-need level 1 | 16,504.0 (46.3) | 14,685 (34.9) |  | 12,210.0 (43.2) | 12,020 (42.5) |  |
| Support-need level 2 | 10,977.0 (30.8) | 13,124 (31.2) |  | 8,868.0 (31.4) | 9,030 (31.9) |  |
| Care-need level 1 | 8,188.0 (23.0) | 14,261 (33.9) |  | 7,194.0 (25.4) | 7,222 (25.5) |  |
| Paralysis, n (%): |  |  | 0.068 |  |  | 0.010 |
| Yes | 12,763.0 (35.8) | 16,436 (39.1) |  | 10,176.0 (36.0) | 10,315 (36.5) |  |
| No | 22,906.0 (64.2) | 25,634 (60.9) |  | 18,096.0 (64.0) | 17,957 (63.5) |  |
| Contractures, n (%): |  |  | 0.032 |  |  | 0.007 |
| Yes | 7,983.0 (22.4) | 9,987 (23.7) |  | 6,417.0 (22.7) | 6,335 (22.4) |  |
| No | 27,686.0 (77.6) | 32,083 (76.3) |  | 21,855.0 (77.3) | 21,937 (77.6) |  |
| Cognitive disorder, n (%): |  |  | 0.088 |  |  | 0.019 |
| Yes | 5,324.0 (14.9) | 7,656 (18.2) |  | 4,556.0 (16.1) | 4,365 (15.4) |  |
| No | 30,345.0 (85.1) | 34,414 (81.8) |  | 23,716.0 (83.9) | 23,907 (84.6) |  |
| Mental or behavioral disorder, n (%): |  |  | 0.119 |  |  | 0.007 |
| Yes | 11,478.0 (32.2) | 15,924 (37.9) |  | 9,419.0 (33.3) | 9,507 (33.6) |  |
| No | 24,191.0 (67.8) | 26,146 (62.1) |  | 18,853.0 (66.7) | 18,765 (66.4) |  |
| Medical care received, n (%): |  |  | 0.067 |  |  | 0.029 |
| Yes | 2,938.0 ( 8.2) | 2,730 ( 6.5) |  | 2,095.0 ( 7.4) | 1,882 ( 6.7) |  |
| No | 32,731.0 (91.8) | 39,340 (93.5) |  | 26,177.0 (92.6) | 26,390 (93.3) |  |
| Urban-rural classification, n (%): |  |  | 0.108 |  |  | — |
| Urban | 15,556.0 (43.6) | 16,170 (38.4) |  | 12,066.0 (42.7) | 12,066 (42.7) |  |
| Intermediate | 11,471.0 (32.2) | 14,405 (34.2) |  | 9,400.0 (33.2) | 9,400 (33.2) |  |
| Rural | 8,642.0 (24.2) | 11,495 (27.3) |  | 6,806.0 (24.1) | 6,806 (24.1) |  |
| Region, n (%): |  |  | 0.176 |  |  | — |
| Hokkaido | 2,036.0 ( 5.7) | 2,403 ( 5.7) |  | 1,687.0 ( 6.0) | 1,687 ( 6.0) |  |
| Tohoku | 1,959.0 ( 5.5) | 2,777 ( 6.6) |  | 1,634.0 ( 5.8) | 1,634 ( 5.8) |  |
| Kanto | 10,188.0 (28.6) | 11,683 (27.8) |  | 8,457.0 (29.9) | 8,457 (29.9) |  |
| Chubu | 4,725.0 (13.2) | 7,423 (17.6) |  | 4,235.0 (15.0) | 4,235 (15.0) |  |
| Kinki | 9,944.0 (27.9) | 9,299 (22.1) |  | 7,093.0 (25.1) | 7,093 (25.1) |  |
| Chugoku/Shikoku | 3,494.0 ( 9.8) | 4,793 (11.4) |  | 3,080.0 (10.9) | 3,080 (10.9) |  |
| Kyushu/Okinawa | 3,323.0 ( 9.3) | 3,692 ( 8.8) |  | 2,086.0 ( 7.4) | 2,086 ( 7.4) |  |

SMD: standardized mean difference.

# Suppl. Table S2. Demographic characteristics of the individuals aged 75–79 before and after the matching of the users and non-users of Japan's long-term care system

|  | **Before propensity score matching** | | | **After propensity score matching** | | |
| --- | --- | --- | --- | --- | --- | --- |
|  | **Non-Users** | **Users** | **SMD** | **Non-users** | **Users** | **SMD** |
| n | 46,606 | 51,205 |  | 35,814 | 35,814 |  |
| Sex, n (%): |  |  | 0.017 |  |  | 0.054 |
| Female | 31,380 (67.3) | 34,886 (68.1) |  | 23,891 (66.7) | 24,797 (69.2) |  |
| Male | 15,226 (32.7) | 16,319 (31.9) |  | 11,923 (33.3) | 11,017 (30.8) |  |
| Support/Care-need level, n (%): |  |  | 0.321 |  |  | 0.004 |
| Support-need level 1 | 22,965 (49.3) | 18,451 (36.0) |  | 15,798 (44.1) | 15,834 (44.2) |  |
| Support-need level 2 | 12,922 (27.7) | 14,176 (27.7) |  | 10,405 (29.1) | 10,344 (28.9) |  |
| Care-need level 1 | 10,719 (23.0) | 18,578 (36.3) |  | 9,611 (26.8) | 9,636 (26.9) |  |
| Paralysis, n (%): |  |  | 0.035 |  |  | 0.017 |
| Yes | 14,050 (30.1) | 16,273 (31.8) |  | 10,743 (30.0) | 11,017 (30.8) |  |
| No | 32,556 (69.9) | 34,932 (68.2) |  | 25,071 (70.0) | 24,797 (69.2) |  |
| Contractures, n (%): |  |  | 0.013 |  |  | 0.001 |
| Yes | 9,370 (20.1) | 10,555 (20.6) |  | 7,132 (19.9) | 7,152 (20.0) |  |
| No | 37,236 (79.9) | 40,650 (79.4) |  | 28,682 (80.1) | 28,662 (80.0) |  |
| Cognitive disorder, n (%): |  |  | 0.170 |  |  | 0.002 |
| Yes | 8,378 (18.0) | 12,773 (24.9) |  | 7,218 (20.2) | 7,247 (20.2) |  |
| No | 38,228 (82.0) | 38,432 (75.1) |  | 28,596 (79.8) | 28,567 (79.8) |  |
| Mental or behavioral disorder, n (%): |  |  | 0.174 |  |  | 0.024 |
| Yes | 17,086 (36.7) | 23,129 (45.2) |  | 13,849 (38.7) | 14,270 (39.8) |  |
| No | 29,520 (63.3) | 28,076 (54.8) |  | 21,965 (61.3) | 21,544 (60.2) |  |
| Medical care received, n (%): |  |  | 0.058 |  |  | 0.002 |
| Yes | 2,532 ( 5.4) | 2,142 ( 4.2) |  | 1,500 ( 4.2) | 1,489 ( 4.2) |  |
| No | 44,074 (94.6) | 49,063 (95.8) |  | 34,314 (95.8) | 34,325 (95.8) |  |
| Urban-rural classification, n (%): |  |  | 0.148 |  |  | — |
| Urban | 19,427 (41.7) | 17,822 (34.8) |  | 14,184 (39.6) | 14,184 (39.6) |  |
| Intermediate | 14,674 (31.5) | 17,127 (33.4) |  | 11,754 (32.8) | 11,754 (32.8) |  |
| Rural | 12,505 (26.8) | 16,256 (31.7) |  | 9,876 (27.6) | 9,876 (27.6) |  |
| Region, n (%): |  |  | 0.200 |  |  | — |
| Hokkaido | 2,504 ( 5.4) | 2,794 ( 5.5) |  | 2,059 ( 5.7) | 2,059 ( 5.7) |  |
| Tohoku | 2,974 ( 6.4) | 4,005 ( 7.8) |  | 2,442 ( 6.8) | 2,442 ( 6.8) |  |
| Kanto | 12,744 (27.3) | 13,800 (27.0) |  | 10,438 (29.1) | 10,438 (29.1) |  |
| Chubu | 6,312 (13.5) | 9,262 (18.1) |  | 5,580 (15.6) | 5,580 (15.6) |  |
| Kinki | 12,495 (26.8) | 10,162 (19.8) |  | 8,115 (22.7) | 8,115 (22.7) |  |
| Chugoku/Shikoku | 4,809 (10.3) | 6,141 (12.0) |  | 4,091 (11.4) | 4,091 (11.4) |  |
| Kyushu/Okinawa | 4,768 (10.2) | 5,041 ( 9.8) |  | 3,089 ( 8.6) | 3,089 ( 8.6) |  |

SMD: standardized mean difference.

# Suppl. Table S3. Demographic characteristics of the individuals aged 80–84 before and after the matching of the users and non-users of Japan's long-term care system

|  | **Before propensity score matching** | | | **After propensity score matching** | | |
| --- | --- | --- | --- | --- | --- | --- |
|  | **Non-Users** | **Users** | **SMD** | **Non-users** | **Users** | **SMD** |
| n | 60,559 | 73,756 |  | 49,043 | 49,043 |  |
| Sex, n (%): |  |  | 0.083 |  |  | 0.079 |
| Female | 39,741 (65.6) | 51,248 (69.5) |  | 32,389.0 (66.0) | 34,190 (69.7) |  |
| Male | 20,818 (34.4) | 22,508 (30.5) |  | 16,654.0 (34.0) | 14,853 (30.3) |  |
| Support/Care-need level, n (%): |  |  | 0.329 |  |  | 0.009 |
| Support-need level 1 | 30,009 (49.6) | 26,608 (36.1) |  | 22,366.0 (45.6) | 22,194 (45.3) |  |
| Support-need level 2 | 16,428 (27.1) | 19,785 (26.8) |  | 13,833.0 (28.2) | 14,016 (28.6) |  |
| Care-need level 1 | 14,122 (23.3) | 27,363 (37.1) |  | 12,844.0 (26.2) | 12,833 (26.2) |  |
| Paralysis, n (%): |  |  | 0.040 |  |  | 0.018 |
| Yes | 16,872 (27.9) | 21,890 (29.7) |  | 13,598.0 (27.7) | 13,989 (28.5) |  |
| No | 43,687 (72.1) | 51,866 (70.3) |  | 35,445.0 (72.3) | 35,054 (71.5) |  |
| Contractures, n (%): |  |  | 0.013 |  |  | 0.006 |
| Yes | 11,967 (19.8) | 14,949 (20.3) |  | 9,755.0 (19.9) | 9,630 (19.6) |  |
| No | 48,592 (80.2) | 58,807 (79.7) |  | 39,288.0 (80.1) | 39,413 (80.4) |  |
| Cognitive disorder, n (%): |  |  | 0.192 |  |  | 0.008 |
| Yes | 11,543 (19.1) | 20,003 (27.1) |  | 10,099.0 (20.6) | 10,264 (20.9) |  |
| No | 49,016 (80.9) | 53,753 (72.9) |  | 38,944.0 (79.4) | 38,779 (79.1) |  |
| Mental or behavioral disorder, n (%): |  |  | 0.190 |  |  | 0.010 |
| Yes | 23,573 (38.9) | 35,619 (48.3) |  | 20,274.0 (41.3) | 20,525 (41.9) |  |
| No | 36,986 (61.1) | 38,137 (51.7) |  | 28,769.0 (58.7) | 28,518 (58.1) |  |
| Medical care received, n (%): |  |  | 0.046 |  |  | 0.020 |
| Yes | 2,567 ( 4.2) | 2,472 ( 3.4) |  | 1,764.0 ( 3.6) | 1,587 ( 3.2) |  |
| No | 57,992 (95.8) | 71,284 (96.6) |  | 47,279.0 (96.4) | 47,456 (96.8) |  |
| Urban-rural classification, n (%): |  |  | 0.165 |  |  | — |
| Urban | 22,246 (36.7) | 21,647 (29.3) |  | 16,662.0 (34.0) | 16,662 (34.0) |  |
| Intermediate | 19,253 (31.8) | 24,601 (33.4) |  | 16,016.0 (32.7) | 16,016 (32.7) |  |
| Rural | 19,060 (31.5) | 27,508 (37.3) |  | 16,365.0 (33.4) | 16,365 (33.4) |  |
| Region, n (%): |  |  | 0.193 |  |  | — |
| Hokkaido | 3,289 ( 5.4) | 3,787 ( 5.1) |  | 2,751.0 ( 5.6) | 2,751 ( 5.6) |  |
| Tohoku | 4,543 ( 7.5) | 6,477 ( 8.8) |  | 4,026.0 ( 8.2) | 4,026 ( 8.2) |  |
| Kanto | 16,177 (26.7) | 18,731 (25.4) |  | 13,788.0 (28.1) | 13,788 (28.1) |  |
| Chubu | 8,999 (14.9) | 14,676 (19.9) |  | 8,294.0 (16.9) | 8,294 (16.9) |  |
| Kinki | 13,994 (23.1) | 12,614 (17.1) |  | 9,433.0 (19.2) | 9,433 (19.2) |  |
| Chugoku/Shikoku | 6,919 (11.4) | 9,474 (12.8) |  | 6,084.0 (12.4) | 6,084 (12.4) |  |
| Kyushu/Okinawa | 6,638 (11.0) | 7,997 (10.8) |  | 4,667.0 ( 9.5) | 4,667 ( 9.5) |  |

SMD: standardized mean difference.

# Suppl. Table S4. Demographic characteristics of the individuals aged ≥85 before and after the matching of the users and non-users of Japan's long-term care system

|  | **Before propensity score matching** | | | **After propensity score matching** | | |
| --- | --- | --- | --- | --- | --- | --- |
|  | **Non-Users** | **Users** | **SMD** | **Non-users** | **Users** | **SMD** |
| n | 54,935 | 74,430 |  | 46,459 | 46,459 |  |
| Sex, n (%): |  |  | 0.115 |  |  | 0.017 |
| Female | 35,489 (64.6) | 52,104 (70.0) |  | 31,387 (67.6) | 31,756 (68.4) |  |
| Male | 19,446 (35.4) | 22,326 (30.0) |  | 15,072 (32.4) | 14,703 (31.6) |  |
| Support/Care-need level, n (%): |  |  | 0.299 |  |  | 0.035 |
| Support-need level 1 | 24,554 (44.7) | 24,243 (32.6) |  | 19,668 (42.3) | 18,878 (40.6) |  |
| Support-need level 2 | 15,171 (27.6) | 19,937 (26.8) |  | 12,683 (27.3) | 13,197 (28.4) |  |
| Care-need level 1 | 15,210 (27.7) | 30,250 (40.6) |  | 14,108 (30.4) | 14,384 (31.0) |  |
| Paralysis, n (%): |  |  | 0.036 |  |  | 0.002 |
| Yes | 14,752 (26.9) | 21,172 (28.4) |  | 12,377 (26.6) | 12,418 (26.7) |  |
| No | 40,183 (73.1) | 53,258 (71.6) |  | 34,082 (73.4) | 34,041 (73.3) |  |
| Contractures, n (%): |  |  | 0.028 |  |  | 0.011 |
| Yes | 10,437 (19.0) | 14,977 (20.1) |  | 8,905 (19.2) | 8,707 (18.7) |  |
| No | 44,498 (81.0) | 59,453 (79.9) |  | 37,554 (80.8) | 37,752 (81.3) |  |
| Cognitive disorder, n (%): |  |  | 0.155 |  |  | 0.005 |
| Yes | 12,434 (22.6) | 21,905 (29.4) |  | 11,149 (24.0) | 11,253 (24.2) |  |
| No | 42,501 (77.4) | 52,525 (70.6) |  | 35,310 (76.0) | 35,206 (75.8) |  |
| Mental or behavioral disorder, n (%): |  |  | 0.171 |  |  | 0.035 |
| Yes | 23,183 (42.2) | 37,738 (50.7) |  | 20,101 (43.3) | 20,909 (45.0) |  |
| No | 31,752 (57.8) | 36,692 (49.3) |  | 26,358 (56.7) | 25,550 (55.0) |  |
| Medical care received, n (%): |  |  | 0.038 |  |  | 0.004 |
| Yes | 1,748 ( 3.2) | 1,902 ( 2.6) |  | 1,226 ( 2.6) | 1,195 ( 2.6) |  |
| No | 53,187 (96.8) | 72,528 (97.4) |  | 45,233 (97.4) | 45,264 (97.4) |  |
| Urban-rural classification, n (%): |  |  | 0.199 |  |  | — |
| Urban | 18,129 (33.0) | 18,352 (24.7) |  | 14,054 (30.3) | 14,054 (30.3) |  |
| Intermediate | 17,578 (32.0) | 24,377 (32.8) |  | 15,100 (32.5) | 15,100 (32.5) |  |
| Rural | 19,228 (35.0) | 31,701 (42.6) |  | 17,305 (37.2) | 17,305 (37.2) |  |
| Region, n (%): |  |  | 0.191 |  |  | — |
| Hokkaido | 2,709 ( 4.9) | 3,641 ( 4.9) |  | 2,347 ( 5.1) | 2,347 ( 5.1) |  |
| Tohoku | 4,116 ( 7.5) | 6,611 ( 8.9) |  | 3,717 ( 8.0) | 3,717 ( 8.0) |  |
| Kanto | 14,952 (27.2) | 18,251 (24.5) |  | 13,037 (28.1) | 13,037 (28.1) |  |
| Chubu | 9,422 (17.2) | 16,969 (22.8) |  | 8,919 (19.2) | 8,919 (19.2) |  |
| Kinki | 10,828 (19.7) | 10,943 (14.7) |  | 7,762 (16.7) | 7,762 (16.7) |  |
| Chugoku/Shikoku | 6,713 (12.2) | 9,679 (13.0) |  | 6,017 (13.0) | 6,017 (13.0) |  |
| Kyushu/Okinawa | 6,195 (11.3) | 8,336 (11.2) |  | 4,660 (10.0) | 4,660 (10.0) |  |

SMD: standardized mean difference.

# Suppl. Table S5. Demographic characteristics of the females before and after the matching of the users and non-users of Japan's long-term care system

|  | **Before propensity score matching** | | | **After propensity score matching** | | |
| --- | --- | --- | --- | --- | --- | --- |
|  | **Non-Users** | **Users** | **SMD** | **Non-users** | **Users** | **SMD** |
| n | 128,755 | 162,954 |  | 109,163 | 109,163 |  |
| Age, yrs, n (%): |  |  | 0.114 |  |  | 0.014 |
| <75 | 22,145 (17.2) | 24,716 (15.2) |  | 17,717 (16.2) | 17,875 (16.4) |  |
| 75–79 | 31,380 (24.4) | 34,886 (21.4) |  | 25,594 (23.4) | 25,281 (23.2) |  |
| 80–84 | 39,741 (30.9) | 51,248 (31.4) |  | 33,717 (30.9) | 34,290 (31.4) |  |
| ≥85 | 35,489 (27.6) | 52,104 (32.0) |  | 32,135 (29.4) | 31,717 (29.1) |  |
| Support/Care-need level, n (%): |  |  | 0.302 |  |  | 0.033 |
| Support-need level 1 | 61,369 (47.7) | 57,421 (35.2) |  | 48,937 (44.8) | 47,242 (43.3) |  |
| Support-need level 2 | 37,401 (29.0) | 47,400 (29.1) |  | 32,390 (29.7) | 33,770 (30.9) |  |
| Care-need level 1 | 29,985 (23.3) | 58,133 (35.7) |  | 27,836 (25.5) | 28,151 (25.8) |  |
| Paralysis, n (%): |  |  | 0.030 |  |  | 0.004 |
| Yes | 37,592 (29.2) | 49,838 (30.6) |  | 32,082 (29.4) | 32,293 (29.6) |  |
| No | 91,163 (70.8) | 113,116 (69.4) |  | 77,081 (70.6) | 76,870 (70.4) |  |
| Contractures, n (%): |  |  | 0.011 |  |  | 0.007 |
| Yes | 27,502 (21.4) | 35,575 (21.8) |  | 23,322 (21.4) | 23,007 (21.1) |  |
| No | 101,253 (78.6) | 127,379 (78.2) |  | 85,841 (78.6) | 86,156 (78.9) |  |
| Cognitive disorder, n (%): |  |  | 0.164 |  |  | 0.002 |
| Yes | 23,918 (18.6) | 41,268 (25.3) |  | 21,550 (19.7) | 21,482 (19.7) |  |
| No | 104,837 (81.4) | 121,686 (74.7) |  | 87,613 (80.3) | 87,681 (80.3) |  |
| Mental or behavioral disorder, n (%): |  |  | 0.174 |  |  | 0.010 |
| Yes | 49,544 (38.5) | 76,651 (47.0) |  | 44,046 (40.3) | 44,565 (40.8) |  |
| No | 79,211 (61.5) | 86,303 (53.0) |  | 65,117 (59.7) | 64,598 (59.2) |  |
| Medical care received, n (%): |  |  | 0.040 |  |  | 0.012 |
| Yes | 4,777 ( 3.7) | 4,871 ( 3.0) |  | 3,492 ( 3.2) | 3,260 ( 3.0) |  |
| No | 123,978 (96.3) | 158,083 (97.0) |  | 105,671 (96.8) | 105,903 (97.0) |  |
| Urban-rural classification, n (%): |  |  | 0.187 |  |  | — |
| Urban | 48,509 (37.7) | 48,022 (29.5) |  | 37,960 (34.8) | 37,960 (34.8) |  |
| Intermediate | 41,243 (32.0) | 54,298 (33.3) |  | 35,536 (32.6) | 35,536 (32.6) |  |
| Rural | 39,003 (30.3) | 60,634 (37.2) |  | 35,667 (32.7) | 35,667 (32.7) |  |
| Region, n (%): |  |  | 0.206 |  |  | — |
| Hokkaido | 6,891 ( 5.4) | 8,526 ( 5.2) |  | 6,210 ( 5.7) | 6,210 ( 5.7) |  |
| Tohoku | 8,999 ( 7.0) | 13,988 ( 8.6) |  | 8,310 ( 7.6) | 8,310 ( 7.6) |  |
| Kanto | 34,702 (27.0) | 41,086 (25.2) |  | 30,775 (28.2) | 30,775 (28.2) |  |
| Chubu | 19,011 (14.8) | 32,616 (20.0) |  | 18,232 (16.7) | 18,232 (16.7) |  |
| Kinki | 30,892 (24.0) | 28,658 (17.6) |  | 22,133 (20.3) | 22,133 (20.3) |  |
| Chugoku/Shikoku | 14,489 (11.3) | 20,781 (12.8) |  | 13,403 (12.3) | 13,403 (12.3) |  |
| Kyushu/Okinawa | 13,771 (10.7) | 17,299 (10.6) |  | 10,100 ( 9.3) | 10,100 ( 9.3) |  |

SMD: standardized mean difference.

# Suppl. Table S6. Demographic characteristics of the males before and after the matching of the users and non-users of Japan's long-term care system

|  | **Before propensity score matching** | | | **After propensity score matching** | | |
| --- | --- | --- | --- | --- | --- | --- |
|  | **Non-Users** | **Users** | **SMD** | **Non-users** | **Users** | **SMD** |
| n | 69,014 | 78,507 |  | 54,795 | 54,795 |  |
| Age, yrs, n (%): |  |  | 0.068 |  |  | 0.051 |
| <75 | 13,524 (19.6) | 17,354 (22.1) |  | 10,559 (19.3) | 11,568 (21.1) |  |
| 75–79 | 15,226 (22.1) | 16,319 (20.8) |  | 12,094 (22.1) | 11,665 (21.3) |  |
| 80–84 | 20,818 (30.2) | 22,508 (28.7) |  | 16,734 (30.5) | 16,005 (29.2) |  |
| ≥85 | 19,446 (28.2) | 22,326 (28.4) |  | 15,408 (28.1) | 15,557 (28.4) |  |
| Support/Care-need level, n (%): |  |  | 0.336 |  |  | 0.007 |
| Support-need level 1 | 32,663 (47.3) | 26,566 (33.8) |  | 23,490 (42.9) | 23,310 (42.5) |  |
| Support-need level 2 | 18,097 (26.2) | 19,622 (25.0) |  | 14,673 (26.8) | 14,698 (26.8) |  |
| Care-need level 1 | 18,254 (26.4) | 32,319 (41.2) |  | 16,632 (30.4) | 16,787 (30.6) |  |
| Paralysis, n (%): |  |  | 0.061 |  |  | 0.031 |
| Yes | 20,845 (30.2) | 25,933 (33.0) |  | 16,286 (29.7) | 17,060 (31.1) |  |
| No | 48,169 (69.8) | 52,574 (67.0) |  | 38,509 (70.3) | 37,735 (68.9) |  |
| Contractures, n (%): |  |  | 0.031 |  |  | 0.001 |
| Yes | 12,255 (17.8) | 14,893 (19.0) |  | 9,734 (17.8) | 9,718 (17.7) |  |
| No | 56,759 (82.2) | 63,614 (81.0) |  | 45,061 (82.2) | 45,077 (82.3) |  |
| Cognitive disorder, n (%): |  |  | 0.163 |  |  | 0.006 |
| Yes | 13,761 (19.9) | 21,069 (26.8) |  | 11,939 (21.8) | 12,074 (22.0) |  |
| No | 55,253 (80.1) | 57,438 (73.2) |  | 42,856 (78.2) | 42,721 (78.0) |  |
| Mental or behavioral disorder, n (%): |  |  | 0.167 |  |  | 0.020 |
| Yes | 25,776 (37.3) | 35,759 (45.5) |  | 21,600 (39.4) | 22,137 (40.4) |  |
| No | 43,238 (62.7) | 42,748 (54.5) |  | 33,195 (60.6) | 32,658 (59.6) |  |
| Medical care received, n (%): |  |  | 0.069 |  |  | 0.002 |
| Yes | 5,008 ( 7.3) | 4,375 ( 5.6) |  | 3,263 ( 6.0) | 3,243 ( 5.9) |  |
| No | 64,006 (92.7) | 74,132 (94.4) |  | 51,532 (94.0) | 51,552 (94.1) |  |
| Urban-rural classification, n (%): |  |  | 0.124 |  |  | — |
| Urban | 26,849 (38.9) | 25,969 (33.1) |  | 20,256 (37.0) | 20,256 (37.0) |  |
| Intermediate | 21,733 (31.5) | 26,212 (33.4) |  | 17,888 (32.6) | 17,888 (32.6) |  |
| Rural | 20,432 (29.6) | 26,326 (33.5) |  | 16,651 (30.4) | 16,651 (30.4) |  |
| Region, n (%): |  |  | 0.174 |  |  | — |
| Hokkaido | 3,647 ( 5.3) | 4,099 ( 5.2) |  | 2,966 ( 5.4) | 2,966 ( 5.4) |  |
| Tohoku | 4,593 ( 6.7) | 5,882 ( 7.5) |  | 3,857 ( 7.0) | 3,857 ( 7.0) |  |
| Kanto | 19,359 (28.1) | 21,379 (27.2) |  | 16,164 (29.5) | 16,164 (29.5) |  |
| Chubu | 10,447 (15.1) | 15,714 (20.0) |  | 9,425 (17.2) | 9,425 (17.2) |  |
| Kinki | 16,369 (23.7) | 14,360 (18.3) |  | 11,136 (20.3) | 11,136 (20.3) |  |
| Chugoku/Shikoku | 7,446 (10.8) | 9,306 (11.9) |  | 6,358 (11.6) | 6,358 (11.6) |  |
| Kyushu/Okinawa | 7,153 (10.4) | 7,767 ( 9.9) |  | 4,889 ( 8.9) | 4,889 ( 8.9) |  |

SMD: standardized mean difference.

# Suppl. Table S7. Demographic characteristics of the individuals with support-need level 1 at baseline before and after the matching of the users and non-users of Japan's long-term care system

|  | **Before propensity score matching** | | | **After propensity score matching** | | |
| --- | --- | --- | --- | --- | --- | --- |
|  | **Non-Users** | **Users** | **SMD** | **Non-users** | **Users** | **SMD** |
| n | 94,032 | 83,987 |  | 71,145 | 71,145 |  |
| Age, yrs, n (%): |  |  | 0.073 |  |  | 0.030 |
| <75 | 16,504 (17.6) | 14,685 (17.5) |  | 12,266 (17.2) | 12,721 (17.9) |  |
| 75–79 | 22,965 (24.4) | 18,451 (22.0) |  | 17,034 (23.9) | 16,275 (22.9) |  |
| 80–84 | 30,009 (31.9) | 26,608 (31.7) |  | 22,673 (31.9) | 23,150 (32.5) |  |
| ≥85 | 24,554 (26.1) | 24,243 (28.9) |  | 19,172 (26.9) | 18,999 (26.7) |  |
| Sex, n (%): |  |  | 0.066 |  |  | 0.037 |
| Female | 61,369 (65.3) | 57,421 (68.4) |  | 46,676 (65.6) | 47,914 (67.3) |  |
| Male | 32,663 (34.7) | 26,566 (31.6) |  | 24,469 (34.4) | 23,231 (32.7) |  |
| Paralysis, n (%): |  |  | 0.026 |  |  | 0.034 |
| Yes | 19,502 (20.7) | 18,305 (21.8) |  | 14,215 (20.0) | 15,206 (21.4) |  |
| No | 74,530 (79.3) | 65,682 (78.2) |  | 56,930 (80.0) | 55,939 (78.6) |  |
| Contractures, n (%): |  |  | 0.014 |  |  | 0.014 |
| Yes | 14,763 (15.7) | 13,605 (16.2) |  | 10,812 (15.2) | 11,170 (15.7) |  |
| No | 79,269 (84.3) | 70,382 (83.8) |  | 60,333 (84.8) | 59,975 (84.3) |  |
| Cognitive disorder, n (%): |  |  | 0.002 |  |  | 0.018 |
| Yes | 5,430 ( 5.8) | 4,882 ( 5.8) |  | 4,213 ( 5.9) | 3,912 ( 5.5) |  |
| No | 88,602 (94.2) | 79,105 (94.2) |  | 66,932 (94.1) | 67,233 (94.5) |  |
| Mental or behavioral disorder, n (%): |  |  | 0.037 |  |  | 0.001 |
| Yes | 18,950 (20.2) | 18,199 (21.7) |  | 14,587 (20.5) | 14,564 (20.5) |  |
| No | 75,082 (79.8) | 65,788 (78.3) |  | 56,558 (79.5) | 56,581 (79.5) |  |
| Medical care received, n (%): |  |  | 0.046 |  |  | 0.017 |
| Yes | 2,012 ( 2.1) | 1,281 ( 1.5) |  | 1,025 ( 1.4) | 882 ( 1.2) |  |
| No | 92,020 (97.9) | 82,706 (98.5) |  | 70,120 (98.6) | 70,263 (98.8) |  |
| Urban-rural classification, n (%): |  |  | 0.181 |  |  | — |
| Urban | 38,418 (40.9) | 27,285 (32.5) |  | 25,785 (36.2) | 25,785 (36.2) |  |
| Intermediate | 29,528 (31.4) | 28,400 (33.8) |  | 23,703 (33.3) | 23,703 (33.3) |  |
| Rural | 26,086 (27.7) | 28,302 (33.7) |  | 21,657 (30.4) | 21,657 (30.4) |  |
| Region, n (%): |  |  | 0.208 |  |  | — |
| Hokkaido | 5,470 ( 5.8) | 5,081 ( 6.0) |  | 4,496 ( 6.3) | 4,496 ( 6.3) |  |
| Tohoku | 5,975 ( 6.4) | 6,781 ( 8.1) |  | 5,195 ( 7.3) | 5,195 ( 7.3) |  |
| Kanto | 24,664 (26.2) | 20,483 (24.4) |  | 18,758 (26.4) | 18,758 (26.4) |  |
| Chubu | 12,773 (13.6) | 14,752 (17.6) |  | 11,540 (16.2) | 11,540 (16.2) |  |
| Kinki | 24,707 (26.3) | 16,030 (19.1) |  | 15,082 (21.2) | 15,082 (21.2) |  |
| Chugoku/Shikoku | 10,678 (11.4) | 11,226 (13.4) |  | 9,158 (12.9) | 9,158 (12.9) |  |
| Kyushu/Okinawa | 9,765 (10.4) | 9,634 (11.5) |  | 6,916 ( 9.7) | 6,916 ( 9.7) |  |

SMD: standardized mean difference.

# Suppl. Table S8. Demographic characteristics of the individuals with support-need level 2 at baseline before and after the matching of the users and non-users of Japan's long-term care system

|  | **Before propensity score matching** | | | **After propensity score matching** | | |
| --- | --- | --- | --- | --- | --- | --- |
|  | **Non-Users** | **Users** | **SMD** | **Non-users** | **Users** | **SMD** |
| n | 55,498 | 67,022 |  | 48,198 | 48,198 |  |
| Age, yrs, n (%): |  |  | 0.064 |  |  | 0.036 |
| <75 | 10,977 (19.8) | 13,124 (19.6) |  | 9,246 (19.2) | 9,673 (20.1) |  |
| 75–79 | 12,922 (23.3) | 14,176 (21.2) |  | 11,294 (23.4) | 10,647 (22.1) |  |
| 80–84 | 16,428 (29.6) | 19,785 (29.5) |  | 14,459 (30.0) | 14,430 (29.9) |  |
| ≥85 | 15,171 (27.3) | 19,937 (29.7) |  | 13,199 (27.4) | 13,448 (27.9) |  |
| Sex, n (%): |  |  | 0.072 |  |  | 0.064 |
| Female | 37,401 (67.4) | 47,400 (70.7) |  | 32,479 (67.4) | 33,905 (70.3) |  |
| Male | 18,097 (32.6) | 19,622 (29.3) |  | 15,719 (32.6) | 14,293 (29.7) |  |
| Paralysis, n (%): |  |  | 0.019 |  |  | 0.031 |
| Yes | 26,345 (47.5) | 32,443 (48.4) |  | 22,502 (46.7) | 23,241 (48.2) |  |
| No | 29,153 (52.5) | 34,579 (51.6) |  | 25,696 (53.3) | 24,957 (51.8) |  |
| Contractures, n (%): |  |  | 0.006 |  |  | 0.012 |
| Yes | 16,887 (30.4) | 20,592 (30.7) |  | 14,821 (30.8) | 14,555 (30.2) |  |
| No | 38,611 (69.6) | 46,430 (69.3) |  | 33,377 (69.2) | 33,643 (69.8) |  |
| Cognitive disorder, n (%): |  |  | 0.006 |  |  | 0.029 |
| Yes | 4,905 ( 8.8) | 5,804 ( 8.7) |  | 4,304 ( 8.9) | 3,917 ( 8.1) |  |
| No | 50,593 (91.2) | 61,218 (91.3) |  | 43,894 (91.1) | 44,281 (91.9) |  |
| Mental or behavioral disorder, n (%): |  |  | 0.022 |  |  | 0.002 |
| Yes | 18,884 (34.0) | 23,510 (35.1) |  | 16,621 (34.5) | 16,671 (34.6) |  |
| No | 36,614 (66.0) | 43,512 (64.9) |  | 31,577 (65.5) | 31,527 (65.4) |  |
| Medical care received, n (%): |  |  | 0.105 |  |  | 0.009 |
| Yes | 5,777 (10.4) | 4,969 ( 7.4) |  | 4,009 ( 8.3) | 3,887 ( 8.1) |  |
| No | 49,721 (89.6) | 62,053 (92.6) |  | 44,189 (91.7) | 44,311 (91.9) |  |
| Urban-rural classification, n (%): |  |  | 0.132 |  |  | — |
| Urban | 20,775 (37.4) | 21,204 (31.6) |  | 17,377 (36.1) | 17,377 (36.1) |  |
| Intermediate | 17,855 (32.2) | 22,171 (33.1) |  | 15,630 (32.4) | 15,630 (32.4) |  |
| Rural | 16,868 (30.4) | 23,647 (35.3) |  | 15,191 (31.5) | 15,191 (31.5) |  |
| Region, n (%): |  |  | 0.188 |  |  | — |
| Hokkaido | 2,722 ( 4.9) | 3,174 ( 4.7) |  | 2,482 ( 5.1) | 2,482 ( 5.1) |  |
| Tohoku | 3,663 ( 6.6) | 5,303 ( 7.9) |  | 3,330 ( 6.9) | 3,330 ( 6.9) |  |
| Kanto | 15,224 (27.4) | 17,372 (25.9) |  | 13,969 (29.0) | 13,969 (29.0) |  |
| Chubu | 8,310 (15.0) | 13,639 (20.4) |  | 7,965 (16.5) | 7,965 (16.5) |  |
| Kinki | 13,633 (24.6) | 12,684 (18.9) |  | 10,537 (21.9) | 10,537 (21.9) |  |
| Chugoku/Shikoku | 6,146 (11.1) | 7,964 (11.9) |  | 5,634 (11.7) | 5,634 (11.7) |  |
| Kyushu/Okinawa | 5,800 (10.5) | 6,886 (10.3) |  | 4,281 ( 8.9) | 4,281 ( 8.9) |  |

SMD: standardized mean difference.

# Suppl. Table S9. Demographic characteristics of the individuals with care-need level 1 at baseline before and after the matching of the users and non-users of Japan's long-term care system

|  | **Before propensity score matching** | | | **After propensity score matching** | | |
| --- | --- | --- | --- | --- | --- | --- |
|  | **Non-Users** | **Users** | **SMD** | **Non-users** | **Users** | **SMD** |
| n | 48,239 | 90,452 |  | 44,768 | 44,768 |  |
| Age, yrs, n (%): |  |  | 0.061 |  |  | 0.029 |
| <75 | 8,188 (17.0) | 14,261 (15.8) |  | 7,515 (16.8) | 7,230 (16.1) |  |
| 75–79 | 10,719 (22.2) | 18,578 (20.5) |  | 9,905 (22.1) | 9,579 (21.4) |  |
| 80–84 | 14,122 (29.3) | 27,363 (30.3) |  | 13,103 (29.3) | 13,515 (30.2) |  |
| ≥85 | 15,210 (31.5) | 30,250 (33.4) |  | 14,245 (31.8) | 14,444 (32.3) |  |
| Sex, n (%): |  |  | 0.044 |  |  | 0.038 |
| Female | 29,985 (62.2) | 58,133 (64.3) |  | 27,866 (62.2) | 28,689 (64.1) |  |
| Male | 18,254 (37.8) | 32,319 (35.7) |  | 16,902 (37.8) | 16,079 (35.9) |  |
| Paralysis, n (%): |  |  | 0.035 |  |  | 0.041 |
| Yes | 12,590 (26.1) | 25,023 (27.7) |  | 11,622 (26.0) | 10,833 (24.2) |  |
| No | 35,649 (73.9) | 65,429 (72.3) |  | 33,146 (74.0) | 33,935 (75.8) |  |
| Contractures, n (%): |  |  | 0.031 |  |  | 0.052 |
| Yes | 8,107 (16.8) | 16,271 (18.0) |  | 7,659 (17.1) | 6,807 (15.2) |  |
| No | 40,132 (83.2) | 74,181 (82.0) |  | 37,109 (82.9) | 37,961 (84.8) |  |
| Cognitive disorder, n (%): |  |  | 0.008 |  |  | 0.030 |
| Yes | 27,344 (56.7) | 51,651 (57.1) |  | 25,392 (56.7) | 26,050 (58.2) |  |
| No | 20,895 (43.3) | 38,801 (42.9) |  | 19,376 (43.3) | 18,718 (41.8) |  |
| Mental or behavioral disorder, n (%): |  |  | 0.011 |  |  | 0.066 |
| Yes | 37,486 (77.7) | 70,701 (78.2) |  | 34,694 (77.5) | 35,904 (80.2) |  |
| No | 10,753 (22.3) | 19,751 (21.8) |  | 10,074 (22.5) | 8,864 (19.8) |  |
| Medical care received, n (%): |  |  | 0.044 |  |  | 0.038 |
| Yes | 1,996 ( 4.1) | 2,996 ( 3.3) |  | 1,549 ( 3.5) | 1,250 ( 2.8) |  |
| No | 46,243 (95.9) | 87,456 (96.7) |  | 43,219 (96.5) | 43,518 (97.2) |  |
| Urban-rural classification, n (%): |  |  | 0.123 |  |  | — |
| Urban | 16,165 (33.5) | 25,502 (28.2) |  | 14,595 (32.6) | 14,595 (32.6) |  |
| Intermediate | 15,593 (32.3) | 29,939 (33.1) |  | 14,062 (31.4) | 14,062 (31.4) |  |
| Rural | 16,481 (34.2) | 35,011 (38.7) |  | 16,111 (36.0) | 16,111 (36.0) |  |
| Region, n (%): |  |  | 0.148 |  |  | — |
| Hokkaido | 2,346 ( 4.9) | 4,370 ( 4.8) |  | 2,302 ( 5.1) | 2,302 ( 5.1) |  |
| Tohoku | 3,954 ( 8.2) | 7,786 ( 8.6) |  | 3,808 ( 8.5) | 3,808 ( 8.5) |  |
| Kanto | 14,173 (29.4) | 24,610 (27.2) |  | 14,014 (31.3) | 14,014 (31.3) |  |
| Chubu | 8,375 (17.4) | 19,939 (22.0) |  | 8,313 (18.6) | 8,313 (18.6) |  |
| Kinki | 8,921 (18.5) | 14,304 (15.8) |  | 7,425 (16.6) | 7,425 (16.6) |  |
| Chugoku/Shikoku | 5,111 (10.6) | 10,897 (12.0) |  | 5,053 (11.3) | 5,053 (11.3) |  |
| Kyushu/Okinawa | 5,359 (11.1) | 8,546 ( 9.4) |  | 3,853 ( 8.6) | 3,853 ( 8.6) |  |

SMD: standardized mean difference.
